# Agonistic anti-CD40 antibodies in patients with solid tumors: a systematic review and evidence map of clinical safety, with exploratory proportional synthesis

**DOI:** 10.3389/fimmu.2026.1908586

**Published:** 2026-08-27

**Authors:** Xinrui Yin, Shijia Du

**Affiliations:** 1 Department of Anesthesiology, Aerospace Center Hospital, Beijing, China; 2 Department of VIP Dental Service, Peking University Stomatological Hospital, Beijing, China

**Keywords:** adverse events, agonistic anti-CD40 antibody, CD40 agonist, cytokine-release syndrome, evidence map, immunotherapy safety, solid tumors, systematic review

## Abstract

**Background:**

Agonistic anti-CD40 antibodies and related CD40 agonist biologics are being developed across multiple solid tumors using diverse biologic formats, delivery routes, and combination regimens. Safety is central to their clinical development but remains difficult to interpret because high-grade adverse events in combination settings may reflect partner therapies, and safety outcomes are reported inconsistently.

**Methods:**

We conducted a systematic review and evidence map of the clinical safety of CD40 agonists in patients with solid tumors. Major bibliographic databases, trial registries, and conference sources were searched. Reports from the same trial population were linked to avoid double counting. Safety-reporting availability was prespecified as a primary output, and exploratory proportional synthesis was performed only when patient-level event counts and denominators were clearly reported or could be reproducibly derived. Clinical activity and immunological or pharmacodynamic findings were summarized descriptively.

**Results:**

Twenty-seven independent peer-reviewed primary safety reports covering 12 agents or biologics were included. The evidence base was uneven and concentrated in conventional systemically administered monoclonal antibodies, basket or mixed-tumor populations, pancreatic cancer, and melanoma. Fc-engineered, tumor-targeted or bispecific, non-monoclonal-antibody, and intratumoral or locally delivered approaches were each represented by few reports, precluding comparison by format or route. Patient-level safety counts and denominators were inconsistently reported, and cytokine-release grading frameworks varied or were unspecified. Several clinically important outcomes were therefore suitable for mapping but not pooling. Four exploratory pools were interpreted as hypothesis-generating: any-grade treatment-related adverse events were consistently frequent but had limited value for distinguishing clinically important toxicity, whereas high-grade and treatment-related estimates were imprecise. In combination studies, pooled estimates reflected regimen-level toxicity rather than toxicity attributable specifically to CD40 agonism. Clinical activity and immunological or pharmacodynamic findings were widely reported but too heterogeneous for formal comparison.

**Conclusion:**

This review provides a structured map of where CD40 agonist safety evidence is available and where important gaps remain. Current evidence does not support definitive comparison of toxicity across biologic formats, delivery routes, or treatment regimens. More consistent patient-level reporting, with explicit denominators, treatment attribution, and grading frameworks, is needed to enable future comparative safety assessment.

**Systematic Review Registration:**

https://www.crd.york.ac.uk/PROSPERO/, identifier CRD420261414658.

## 1 Introduction

CD40 is a costimulatory receptor expressed mainly on antigen-presenting cells, including dendritic cells, macrophages, and B cells. Engagement of CD40 by CD40 ligand (CD40L) activates these cells, increases costimulatory signaling and cytokine production, and promotes T-cell priming, providing a biological rationale for therapeutic CD40 agonism in cancer immunotherapy (1–3). In contrast to immune checkpoint inhibitors (ICIs), which act largely by relieving inhibitory signals on T cells, CD40 agonists are intended to strengthen antigen presentation and initiate or amplify antitumor immune responses (4). This rationale has supported the clinical development of agonistic anti-CD40 antibodies and related CD40 agonist biologics across multiple solid tumors (5–9). Clinical development has expanded beyond conventional systemically administered monoclonal antibodies (mAbs) to include Fc-engineered or Fc-optimized antibodies, tumor-targeted or bispecific constructs, intratumoral or locally delivered approaches, and non-mAb biologics (5–7, 9). These agents have been evaluated as monotherapy and, more commonly, in combination with chemotherapy, ICIs, radiotherapy or chemoradiotherapy, vaccines, and other anticancer strategies (8–15). This diversity broadens the therapeutic scope of CD40 agonism but complicates safety interpretation. Antibody format, Fc gamma receptor (FcγR) engagement, delivery route, dose and schedule, tumor setting, and combination partner may influence immune activation and toxicity, while high-grade adverse events in combination regimens may also reflect the contribution of partner therapies (5–7, 11, 12).

Safety assessment is therefore central to interpreting the CD40 agonist literature. Early clinical studies have reported cytokine-release reactions, infusion-related or hypersensitivity reactions, hepatic laboratory abnormalities, constitutional symptoms, serious adverse events, treatment discontinuation, treatment-related deaths, and dose-limiting toxicities (7, 8, 10–12, 14, 15). The evidence base is predominantly composed of early-phase, open-label, dose-escalation, dose-expansion, or basket studies with heterogeneous regimens and relatively small safety-evaluable populations (9–15). These studies also report clinical activity and immunological or pharmacodynamic findings, but substantial variation in endpoints, sampling methods, assessment times, and patient populations limits formal cross-study comparison.

Interpretation is further constrained by differences in safety reporting. Quantitative synthesis requires clear reporting of the number of patients who experienced an event and the number evaluated, whether events were considered treatment related, how severity was graded, and whether multiple reports arose from overlapping trial populations (16, 17). Per-event adverse-event listings cannot be converted directly into the number of affected patients, and dose-limiting toxicities cannot be assumed to represent the overall burden of treatment-related grade ≥3 adverse events. A single pooled estimate may therefore be misleading if it combines all-cause and treatment-related outcomes, monotherapy and combination regimens, grade ≥3 and any-grade events, or patient-level and event-level data.

Accordingly, we conducted a systematic review and evidence map of agonistic anti-CD40 antibodies and related CD40 agonist biologics in patients with solid tumors. We aimed to characterize the clinical safety evidence by agent, format, route, tumor setting, and treatment context; assess the completeness and consistency of safety reporting; identify overlapping trial reports and non-independent patient populations; and distinguish outcomes that could and could not be quantitatively summarized. We also descriptively summarized the reported clinical activity and immunological or pharmacodynamic context of the included trials without undertaking efficacy or immunological/pharmacodynamic meta-analysis. Exploratory proportional synthesis was performed only where comparable patient-level data were directly reported or could be reproducibly derived. The review was designed to clarify the current safety evidence, its reporting limitations, and the priorities needed to support future comparative assessment.

## 2 Methods

### 2.1 Protocol registration and reporting framework

This systematic review was prospectively registered in PROSPERO (CRD420261414658) on 3 June 2026 and amended on 9 June 2026. The registration record is publicly accessible in the PROSPERO database under the title “Agonistic anti-CD40 antibodies in patients with solid tumors: a systematic review and evidence map of clinical safety, with exploratory proportional synthesis.” Reporting followed the Preferred Reporting Items for Systematic Reviews and Meta-Analyses (PRISMA) 2020 statement (18).

The original protocol planned a stratified proportional meta-analysis of clinical safety outcomes by antibody format and delivery route. During full-text and supplementary-material extraction, patient-level grade ≥3 treatment-related adverse events were found to be inconsistently reported across early-phase studies. The planned stratification was also not feasible because comparable patient-level safety data were unavailable in key format and route strata, particularly for systemically administered Fc-engineered antibodies. The review was therefore amended to a systematic review and evidence map with exploratory proportional synthesis. The core search strategy, eligibility criteria, safety-related data-extraction framework, and duplicate-report handling rules remained unchanged. Exploratory proportional synthesis was undertaken only where studies provided an explicit denominator and either a directly reported or reproducibly derived patient-level numerator for the relevant outcome. All pooled estimates were interpreted as hypothesis-generating rather than as definitive estimates of class-level toxicity.

### 2.2 Eligibility criteria

Eligible studies were clinical studies enrolling adult patients with histologically or cytologically confirmed solid tumors who received an agonistic anti-CD40 antibody or related CD40 agonist biologic, as monotherapy or in combination with chemotherapy, immune checkpoint inhibitors (ICIs), radiotherapy, vaccines, or other anticancer agents. All tumor types and treatment settings (advanced, metastatic, unresectable, recurrent, neoadjuvant, or adjuvant) were eligible. Mixed-tumor studies were eligible when solid-tumor safety data were reported separately or the population was predominantly solid-tumor. Eligible interventions comprised conventional anti-CD40 monoclonal antibodies (mAbs), Fc-engineered or Fc-optimized anti-CD40 antibodies, tumor-targeted or bispecific CD40 agonists, intratumoral or locally delivered approaches, and non-mAb CD40 agonists (e.g., designed ankyrin repeat protein [DARPin] constructs). No comparator was required; for comparative or randomized studies, only the CD40-containing arm was used.

Eligible designs included phase I/Ib/II, dose-escalation, dose-expansion, basket, neoadjuvant, and other prospective clinical studies reporting safety after a CD40 agonist. Published articles, conference reports, registry records, and linked supplementary or translational reports were considered when they provided extractable clinical safety data or clarified trial identity, treatment context, safety denominators, or duplicate reporting. Conference abstracts and registry records were included in the evidence map when they contributed safety information or trial identification, but were not used as safety denominators when they provided only background or mechanistic information or lacked extractable clinical safety data. We excluded preclinical, animal, and *in vitro* studies; reviews, editorials, and commentaries; non-agonistic or non-CD40 interventions; studies restricted to hematological malignancies; and studies restricted to pediatric populations.

### 2.3 Information sources and search strategy

PubMed/MEDLINE, Embase, Web of Science Core Collection, Scopus, and the Cochrane Central Register of Controlled Trials (CENTRAL) were searched on 3 June 2026. Additional searches were conducted on the same day in ClinicalTrials.gov and were supplemented by targeted Google Scholar searches, conference sources from the American Society of Clinical Oncology (ASCO), American Association for Cancer Research (AACR), and Society for Immunotherapy of Cancer (SITC), and reference-list screening of relevant reviews and reports. Full search strategies are provided in **Supplementary Table 1** (19).

The strategy combined class-level terms for CD40 agonism with individual agent names to improve sensitivity. Class-level terms included “CD40 agonist,” “agonistic CD40,” “anti-CD40 agonist,” and “CD40 agonistic antibody.” Individual agent names included CP-870,893, selicrelumab/RO7009789, sotigalimab/APX005M, mitazalimab/ADC-1013/JNJ-64457107, SEA-CD40, CDX-1140, ChiLob 7/4, 2141-V11, RO7300490, ABBV-428, SL-172154, LVGN7409, and MP0317. These agent-specific terms supplemented the broader class-level terms and were not intended to constitute an exhaustive list of all development codes or aliases. The terms were combined with cancer and clinical or safety terms according to the syntax of each database, with non-human records removed where applicable. In ClinicalTrials.gov, each agent name was additionally searched as a broad term, including dacetuzumab/SGN-40; agents evaluated exclusively in hematological malignancies were removed during study selection. Restriction to solid-tumor clinical records evaluating CD40 agonists was applied during screening rather than within the search string.

A targeted alias verification was performed using the original eligibility criteria and the original search cutoff of 3 June 2026. RG7876 was confirmed as a development code for selicrelumab and was already covered by the terms selicrelumab, CP-870,893, and RO7009789. Giloralimab, also known as ABBV-927, was verified using the same criteria and cutoff. When multiple sources referred to the same trial, records were cross-checked using registration numbers, antibody names and aliases, titles, authors, publication years, tumor types, treatment regimens, and patient populations to support trial-family linkage.

### 2.4 Study selection, record linkage, and duplicate-report handling

Records were imported and deduplicated at the citation level before title and abstract screening. Two reviewers independently screened titles and abstracts against the eligibility criteria, followed by independent full-text assessment of potentially relevant records. Disagreements were resolved through discussion and consensus. Linkage of multiple reports arising from the same trial population was handled separately at the trial-family level.

Full-text reports were assessed together with available appendices, registry entries, conference reports, and linked translational, biomarker, or pharmacodynamic reports. Records describing the same or overlapping study population were linked rather than treated as independent studies. Linked records were used to clarify trial identity, intervention characteristics, safety definitions, safety-evaluable denominators, outcome availability, and patient overlap. Translational or biomarker reports were not counted as independent safety reports unless they described a distinct clinical safety population.

To avoid double counting, each trial family contributed only one non-overlapping safety denominator to any denominator-based evidence-map summary or exploratory synthesis of a given outcome. Priority was determined separately for each outcome according to the availability of a non-overlapping patient-level numerator and denominator, rather than solely by report recency or sample size. When multiple reports provided comparable data for the same outcome and population, the most complete peer-reviewed report was used. Earlier preprints, conference reports, registry records, and linked reports were retained for trial identification, evidence mapping, supplementary information, or overlap clarification unless they provided a distinct eligible clinical population. Reasons for exclusion or non-use as an independent denominator were documented, and trial-family linkage and overlap assessments are detailed in **Supplementary Table 3**.

### 2.5 Data extraction and safety-reporting availability

Two reviewers independently extracted data using a structured form and cross-checked the extracted information against the source reports. Discrepancies were resolved by consensus. Appendices, registry records, and linked reports were consulted when an outcome or denominator was incompletely reported in the primary publication.

Extraction covered study characteristics (author, year, source, publication type, phase, design, tumor type, treatment setting, registration number, enrolled population, and safety-evaluable population); intervention characteristics (antibody or biologic name and aliases, biologic format, immunoglobulin subclass and Fc modification, route, dose and schedule, treatment context, and combination partner); safety outcomes; and the availability and form of safety reporting.

Safety outcomes comprised all-cause grade ≥3 treatment-emergent adverse events (TEAEs); investigator-reported treatment-related grade ≥3 adverse events (TRAEs); any-grade TRAEs; cytokine-release reactions; infusion-related or hypersensitivity reactions; hepatic toxicity; serious adverse events (SAEs); treatment discontinuation due to toxicity; treatment-related deaths; and dose-limiting toxicities (DLTs). Patient-level numerators and denominators were extracted where reported. To preserve the distinction between attribution and severity, all-cause and treatment-related events were recorded separately. Grade ≥3 events were not inferred from DLT definitions or from non-grade-specific treatment-related event reports. Per-event or preferred-term listings were not summed to create patient-level counts because an individual patient could experience more than one event. We also recorded the Common Terminology Criteria for Adverse Events (CTCAE) version, the basis used to assign treatment relatedness, and, for cytokine-release reactions, the grading framework applied (American Society for Transplantation and Cellular Therapy [ASTCT] 2019, Lee 2014, CTCAE, or unspecified) (20–22).

Because high-grade safety outcomes were expected to be reported inconsistently, the availability and form of safety reporting were treated as a prespecified analytical dimension. For each study and outcome, reporting was categorized as an explicit patient-level numerator and denominator; a percentage without a directly tabulated count; an all-cause figure without treatment-related attribution; a treatment-related figure without grade-specific patient-level data; a per-event or preferred-term listing without a patient-level any-event count; an unclear denominator; or not reported. This reporting-availability map was a primary output of the review and determined which outcomes were eligible for exploratory synthesis. The full study-level matrix is provided in **Supplementary Table 5**.

Clinical activity endpoints (objective response rate, disease control rate, progression-free survival, overall survival, pathologic response, and other study-defined activity outcomes) and immunological or pharmacodynamic findings (antigen-presenting-cell or dendritic-cell activation, B- and T-cell changes, cytokine or chemokine responses, receptor occupancy, peripheral-blood findings, paired tumor-biopsy or tumor microenvironment findings, and pretreatment immune-related measurements) were recorded descriptively as reported. Information from linked translational or biomarker reports was integrated into the corresponding trial family and was not counted as an additional independent study. Pretreatment immune-related measurements were distinguished from on-treatment pharmacodynamic changes. No efficacy or immunological/pharmacodynamic meta-analysis or formal cross-study comparison was performed, and no causal relationship between immunological changes and adverse events was inferred. Study-level clinical activity and immunological/pharmacodynamic context are presented in **Supplementary Tables 6, S7**, respectively.

### 2.6 Classification of antibody format, delivery route, and treatment context

Each agent was classified before evidence mapping or synthesis. Biologic format was categorized as conventional anti-CD40 mAb, Fc-engineered or Fc-optimized antibody, tumor-targeted or bispecific CD40 agonist, non-mAb CD40 agonist, or unclear/other. Non-mAb biologics, including DARPin-based constructs and Fc-fusion proteins, and tumor-targeted or bispecific agents were retained as distinct format strata and were not combined with conventional mAbs except in explicitly labeled exploratory analyses.

Delivery route was categorized as systemic (intravenous or subcutaneous), intratumoral or local, or unclear. Intratumoral or locally delivered agents were mapped separately because the route of administration may alter exposure patterns and the interpretation of systemic and local toxicity. Treatment context was categorized as monotherapy or combination therapy, with combinations classified according to the principal partner as chemotherapy, ICI, chemotherapy plus ICI, radiotherapy or chemoradiotherapy, vaccine-based therapy, or other combination therapy. Where a study used multiple routes, schedules, cohorts, or treatment partners, classification and data extraction were matched to the safety-evaluable population for the relevant outcome. These classifications structured the evidence map and determined whether exploratory synthesis was appropriate within clinically comparable strata.

### 2.7 Safety outcomes and exploratory synthesis pools

The primary analytical focus was the clinical safety evidence map and the availability and consistency of high-grade safety reporting, rather than estimation of a single pooled toxicity rate. Within this framework, investigator-reported treatment-related grade ≥3 adverse events were retained as the attribution-specific outcome of primary interest, consistent with the original protocol. Cytokine-release reactions, infusion-related or hypersensitivity reactions, hepatic toxicity, serious adverse events, treatment discontinuation due to toxicity, treatment-related deaths, and dose-limiting toxicities were summarized descriptively and were considered for quantitative exploration only when comparable patient-level data were available.

Exploratory proportional synthesis used four prespecified outcome pools designed to maintain explicit distinctions in event attribution and treatment context. Pool 1 comprised all-cause grade ≥3 TEAEs in monotherapy studies and was defined as the monotherapy high-grade anchor, although its all-cause definition did not isolate events attributable to CD40 agonism. Pool 2 comprised all-cause grade ≥3 TEAEs in combination-therapy studies and summarized the high-grade toxicity burden of the complete treatment regimen rather than toxicity specifically attributable to the CD40 agonist. Pool 3 comprised investigator-reported treatment-related grade ≥3 adverse events within the conventional or Fc-engineered systemic antibody stratum; investigator-assigned relatedness in combination studies could not be attributed exclusively to the CD40 agonist. Pool 4 comprised any-grade TRAEs and described the overall frequency of treatment-attributed adverse events irrespective of severity.

Each pool included only studies with an explicit denominator and either a directly reported or reproducibly derived patient-level numerator for the relevant outcome. Derived values were accepted only when the denominator was explicit and the calculation could be reproduced from the source report. Per-event listings, unclear denominators, DLT counts used as substitutes for treatment-related grade ≥3 adverse events, and non-grade-specific treatment-related summaries were not eligible. The originally planned comparison by antibody format and delivery route was retained as an analytical aim but was conditional on the availability of comparable patient-level data within each stratum. Cytokine-release reactions were not pooled because grading frameworks varied or were unspecified; they were summarized descriptively, and differences in grading systems were treated as an important source of clinical and reporting heterogeneity.

### 2.8 Study quality assessment

Study quality was assessed using the Methodological Index for Non-Randomized Studies (MINORS), selected because the evidence base consisted predominantly of early-phase, open-label, single-arm, dose-escalation, dose-expansion, and other non-randomized studies (23). For each independent primary safety report, the eight non-comparative MINORS items were assessed: clearly stated aim; inclusion of consecutive patients; prospective data collection; endpoints appropriate to the study aim; unbiased endpoint assessment; follow-up appropriate to the study aim; loss to follow-up below 5%; and prospective sample-size calculation. Each item was scored as 0 (not reported), 1 (reported but inadequate), or 2 (reported and adequate), for a maximum score of 16. The Cochrane Risk of Bias 2 (RoB 2) tool was not applied because the review did not estimate randomized between-arm treatment effects. Reporting features relevant to interpretability and poolability, including clarity of the safety-evaluable denominator, treatment attribution, CTCAE version, availability of patient-level data, cytokine-release grading, and overlapping reports, were recorded separately and were not incorporated into the MINORS score. Study quality was used to contextualize reliability and reporting transparency and was not used to exclude studies. Per-study MINORS assessments are provided in **Supplementary Table 4**.

Potential bias arising from study selection, data extraction, duplicate reporting, treatment attribution, and incomplete outcome reporting was addressed through independent dual screening and extraction, consensus resolution of disagreements, trial-family linkage, and use of one non-overlapping denominator per trial family and outcome. The most complete peer-reviewed publication was prioritized when an earlier preprint, conference report, or overlapping publication described the same population. All-cause and treatment-related outcomes were kept separate, DLTs were not used as substitutes for grade ≥3 TRAEs, and per-event listings were not summed into patient-level any-event counts. Values derived from reported percentages or narrative statements were identified in the study-level dataset and examined in sensitivity analyses that excluded derived values.

### 2.9 Evidence mapping and exploratory proportional synthesis

The primary synthesis was an evidence map of clinical safety. Studies were mapped according to agent, biologic format, delivery route, tumor type, treatment setting and context, combination partner, and safety-outcome reporting availability. Reporting availability was summarized using structured tables and heatmaps. Study-level safety proportions were displayed when explicit patient-level data were available, and reasons for non-poolability were documented otherwise. Study-level 95% confidence intervals were calculated using the Clopper–Pearson exact method. Narrative synthesis was used for safety outcomes or strata unsuitable for pooling, including cytokine-release reactions and, unless comparable patient-level data were available in a sufficiently homogeneous set, serious adverse events, hepatic toxicity, infusion-related or hypersensitivity reactions, treatment discontinuation due to toxicity, treatment-related deaths, and dose-limiting toxicities. Clinical activity and immunological/pharmacodynamic findings were presented separately as descriptive contextual information and were not quantitatively synthesized.

Exploratory proportional synthesis was performed only for pools or strata containing at least four studies with comparable directly reported or reproducibly derived patient-level numerator and denominator data. Analyses used R version 4.6.0 and the meta package, version 8.3.0; single-arm proportions were pooled using metaprop (24). The primary model was an inverse-variance random-effects model applied to logit-transformed proportions, with restricted maximum likelihood estimation of between-study variance and Hartung–Knapp adjustment. Pooled proportions were reported with 95% confidence intervals and, where feasible, 95% prediction intervals; statistical heterogeneity was described using I² and τ² (25). A continuity correction of 0.5 was applied to studies reporting event proportions of 0% or 100% when required for the logit transformation.

Sensitivity analyses comprised the Freeman–Tukey double-arcsine transformation, a fixed-effect descriptive analysis, a generalized linear mixed model with a binomial likelihood and logit link without continuity correction as a boundary-study check, and exclusion of values derived from reported percentages or narrative safety statements. Derived values were retained only when the denominator was explicit and the derivation reproducible and were identified as approximate or text-derived in the study-level dataset. Funnel plots and formal tests for small-study effects were not performed because all exploratory pools contained fewer than 10 studies.

All pooled proportions were considered exploratory and hypothesis-generating. They were not interpreted as definitive class-level toxicity rates when heterogeneity was substantial, confidence intervals were very wide, patient-level reporting was incomplete, or event attribution was unclear. Combination-regimen pools were interpreted as summaries of regimen-level toxicity burden rather than as toxicity attributable specifically to CD40 agonism. Strata lacking sufficient comparable data were reported narratively within the evidence map. Forest plots, prediction intervals, and sensitivity analyses are provided in **Supplementary Figure 1**.

## 3 Results

### 3.1 Study selection, trial linkage, and evidence base

**Figure 1:**
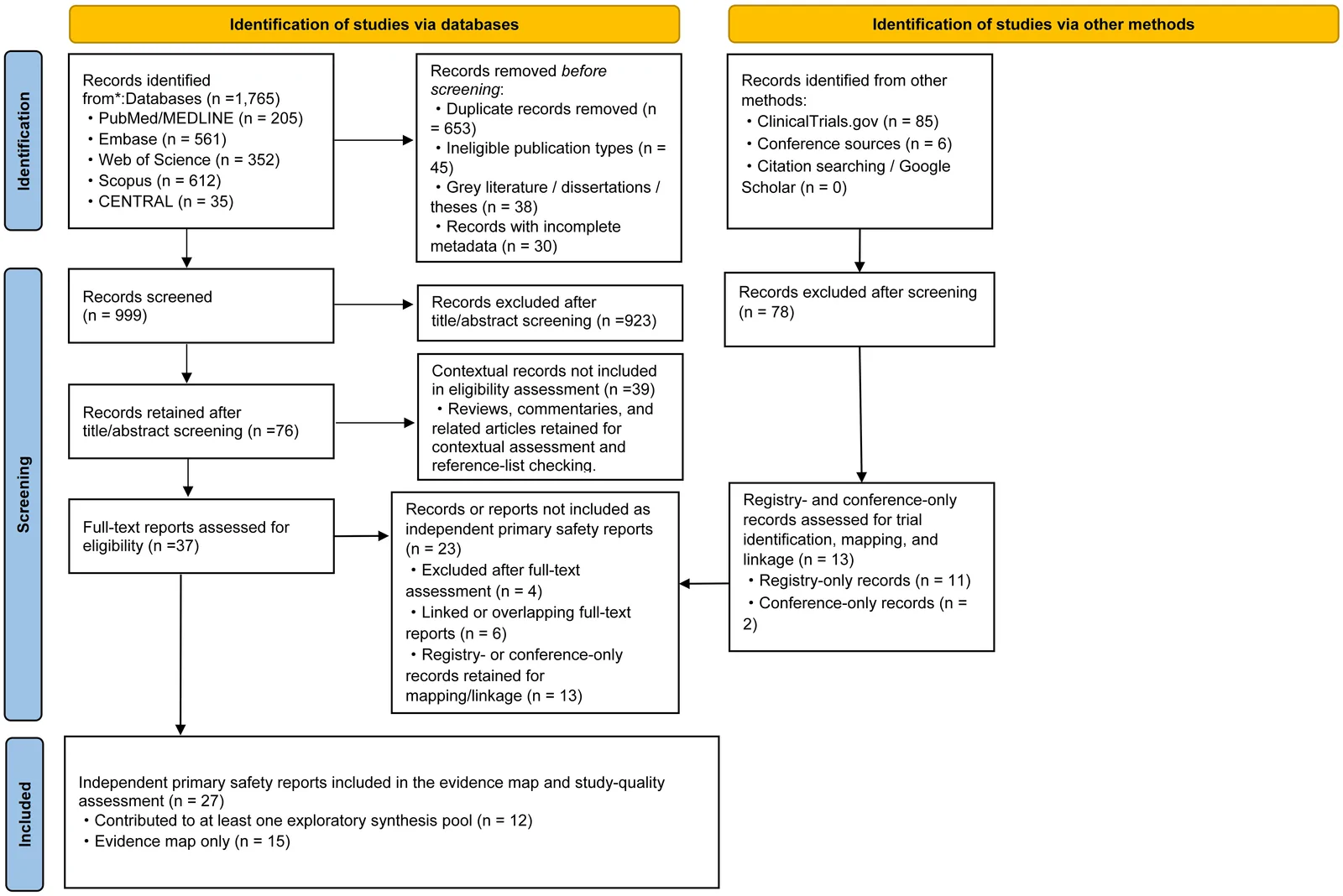
PRISMA 2020 flow diagram of study selection and construction of the final evidence base. Records were identified from five databases, ClinicalTrials.gov, conference sources, and supplementary searching. Linked reports were assigned to trial families and were not counted as additional independent safety reports. Registry- and conference-only records were used for trial mapping but did not contribute independent denominators. The final evidence base comprised 27 independent primary safety reports; 12 contributed to at least one exploratory proportional-synthesis pool and 15 to the evidence map only. PRISMA, Preferred Reporting Items for Systematic Reviews and Meta-Analyses.

The electronic database searches identified 1,765 records: 205 from PubMed/MEDLINE, 561 from Embase, 352 from Web of Science Core Collection, 612 from Scopus, and 35 from the Cochrane Central Register of Controlled Trials. Before screening, 766 records were removed, comprising 653 duplicates, 45 ineligible publication types, 38 grey-literature, dissertation, or thesis records, and 30 records with incomplete metadata. The remaining 999 unique records underwent title and abstract screening, of which 923 were excluded. Of the 76 records retained, 37 full-text reports were retrieved and assessed for eligibility, while 39 reviews, commentaries, and related articles were retained only for contextual assessment and reference-list checking and did not enter the eligibility assessment (**Figure 1**).

Searches conducted through other methods identified 91 additional records: 85 from ClinicalTrials.gov and six from conference sources; no additional records were identified through citation searching or Google Scholar. After screening, 78 records were excluded. The remaining 13 records, comprising 11 registry-only and two conference-only records, were assessed for trial identification, evidence mapping, and trial-family linkage.

Across both identification pathways, four reports were excluded after full-text assessment, six linked or overlapping reports were not retained as independent primary safety reports, and the 13 registry-only or conference-only records were retained for mapping or linkage only. None of the latter records contributed an independent safety denominator or entered an exploratory synthesis pool. The final evidence base therefore comprised 27 independent primary safety reports, all represented by peer-reviewed full-text publications. One study of the intratumoral Fc-optimized antibody 2141-V11, initially identified through a Research Square preprint, was represented in the final evidence base by its subsequent peer-reviewed Cancer Cell publication. Targeted verification of RG7876 and giloralimab/ABBV-927 identified no additional eligible independent primary safety report and did not alter the evidence base.

The 27 independent reports formed the basis of the study-quality assessment and safety evidence map. Twelve provided a clear denominator and a directly reported or reproducibly derived patient-level event count suitable for at least one exploratory proportional-synthesis pool; the remaining 15 contributed to the evidence map only.

Trial-family linkage was used to prevent overlapping populations from contributing more than one safety denominator for the same outcome. The PRINCE trial (NCT03214250) evaluated sotigalimab-based regimens in metastatic pancreatic ductal adenocarcinoma across linked phase 1b and phase 2 reports. Because the phase 2 safety population incorporated the phase 1b lead-in cohort, the reports were treated as a single trial family. The phase 1b report was retained where it provided non-overlapping, outcome-specific patient-level data, whereas the phase 2 report was treated as a linked report and did not contribute an additional independent denominator. Trial-family linkage and overlap assessments for all trial families are detailed in **Supplementary Table 3**.

### 3.2 Landscape of CD40 agonist clinical studies

**Figure 2:**
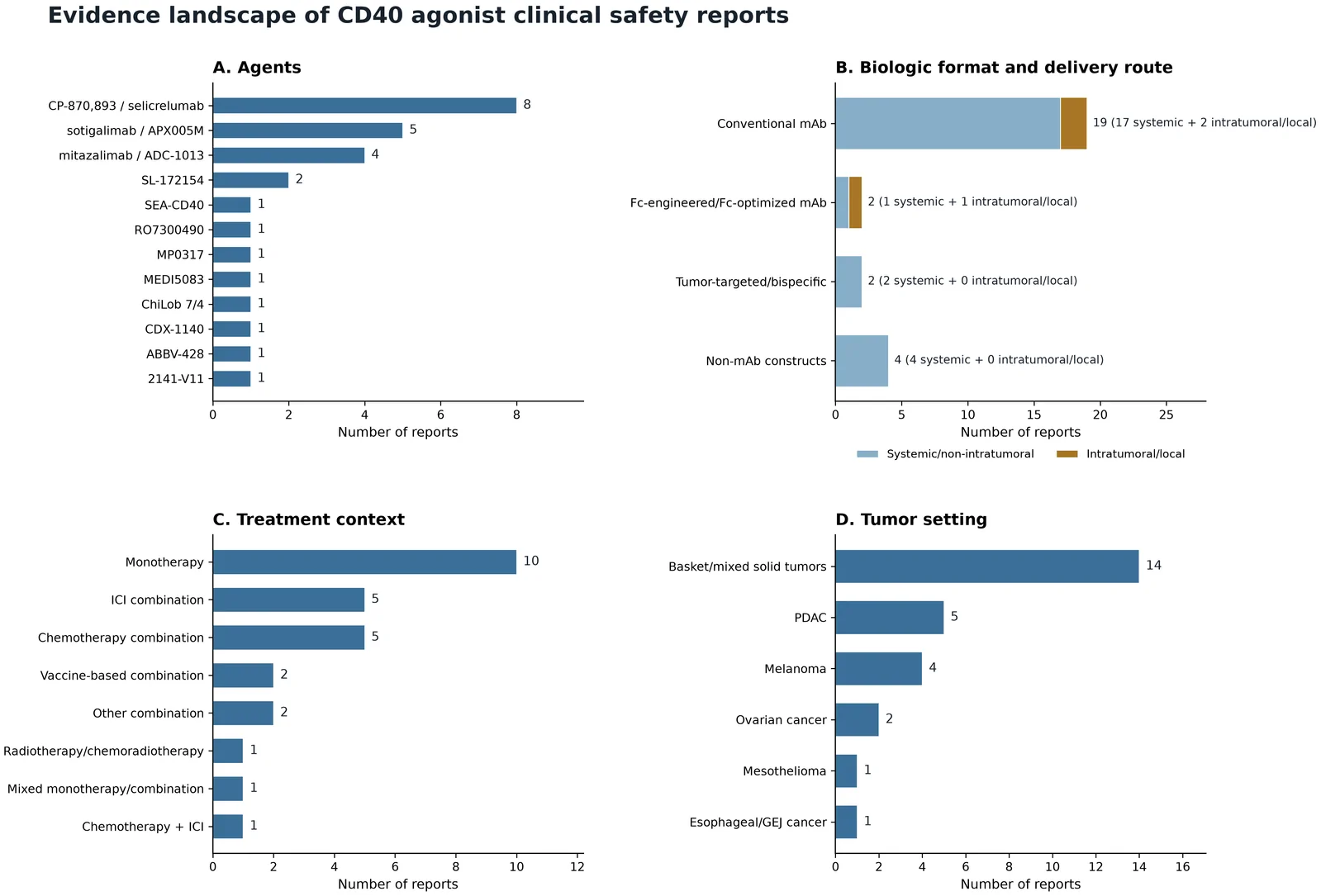
Evidence landscape of CD40 agonist clinical safety reports. Distribution of the 27 independent primary safety reports by **(A)** agent or biologic, **(B)** biologic format and delivery route, **(C)** treatment context, and **(D)** dominant tumor setting. Agent aliases were consolidated, and counts sum to 27 in each panel. Systemic/non-intratumoral administration included peripheral subcutaneous or intradermal delivery. DARPin, designed ankyrin repeat protein; GEJ, gastroesophageal junction; ICI, immune checkpoint inhibitor; mAb, monoclonal antibody; PDAC, pancreatic ductal adenocarcinoma.

The 27 independent primary safety reports covered 12 CD40 agonist agents or biologics (**Table 1**; **Figure 2**), with full study-level characteristics provided in **Supplementary Table 2**. The most frequently represented agents were CP-870,893/selicrelumab (eight reports), sotigalimab/APX005M (five reports), and mitazalimab/ADC-1013 (four reports), followed by SL-172154 (two reports); each of the remaining eight agents was represented by a single report. The evidence base was predominantly composed of early-phase dose-escalation, dose-expansion, basket, or tumor-specific studies with heterogeneous treatment settings and safety-reporting structures.

**Table 1:** Characteristics of the 27 independent primary safety reports included in the CD40 agonist safety evidence map.

Study	Agent	Format	Route	Treatment context	Tumor type	Safety-evaluable N	Exploratory pool
Ninmer et al., 2026 (26)	CDX-1140	Conventional mAb	Systemic (subcutaneous/intradermal administration)	Vaccine-based combination (melanoma helper peptide vaccine)	Melanoma	22	Yes (Pools 2/3/4)
Johnson et al., 2015 (27)	ChiLob 7/4	Conventional mAb	Systemic	Monotherapy	Basket/mixed solid tumors	28	No
Vonderheide et al., 2007 (10)	CP-870,893/selicrelumab	Conventional mAb	Systemic	Monotherapy	Basket/mixed solid tumors	29	No
Rüter et al., 2010 (28)	CP-870,893/selicrelumab	Conventional mAb	Systemic	Monotherapy	Basket/mixed solid tumors	27	No
Beatty et al., 2013 (29)	CP-870,893/selicrelumab	Conventional mAb	Systemic	Chemotherapy combination (gemcitabine)	PDAC	21	No
Vonderheide et al., 2013 (8)	CP-870,893/selicrelumab	Conventional mAb	Systemic	Chemotherapy combination (carboplatin)	Basket/mixed solid tumors	32	No
Nowak et al., 2015 (30)	CP-870,893/selicrelumab	Conventional mAb	Systemic	Chemotherapy combination (cisplatin + pemetrexed)	Mesothelioma	15	No
Bajor et al., 2018 (31)	CP-870,893/selicrelumab	Conventional mAb	Systemic	ICI combination (tremelimumab)	Melanoma	24	No
Machiels et al., 2020 (32)	CP-870,893/selicrelumab	Conventional mAb	Systemic	Other combination (emactuzumab)	Basket/mixed solid tumors	37	Yes (Pools 2/3)
Byrne et al., 2021 (33)	CP-870,893/selicrelumab	Conventional mAb	Systemic	Chemotherapy combination (gemcitabine + nab-paclitaxel)	PDAC	16	No
Irenaeus et al., 2019 (34)	mitazalimab/ADC-1013	Conventional mAb	Intratumoral/local	Monotherapy	Basket/mixed solid tumors	23	No
Moreno et al., 2023 (15)	mitazalimab/ADC-1013	Conventional mAb	Systemic	Monotherapy	Basket/mixed solid tumors	95	Yes (Pools 1/4)
Van Laethem et al., 2024 (12)	mitazalimab/ADC-1013	Conventional mAb	Systemic	Chemotherapy combination (mFOLFIRINOX)	PDAC	70	Yes (Pools 2/4)
Kucukcelebi et al., 2025 (35)	mitazalimab/ADC-1013	Conventional mAb	Systemic	Vaccine-based combination (MesoPher autologous dendritic cell vaccine)	PDAC	16	Yes (Pools 3/4)
O’Hara et al., 2021 (11)	sotigalimab/APX005M	Conventional mAb	Systemic	Chemotherapy with or without ICI combination (gemcitabine plus nab-paclitaxel, with or without nivolumab)	PDAC	30	Yes (Pools 3/4)
Weiss et al., 2021 (36)	sotigalimab/APX005M	Conventional mAb	Systemic	Cabiralizumab-based combination, with or without nivolumab	Basket/mixed solid tumors	26	Yes (Pool 4)
Weiss et al., 2024 (37)	sotigalimab/APX005M	Conventional mAb	Systemic	ICI combination (nivolumab)	Melanoma	38	Yes (Pools 2/3)
Ko et al., 2025 (38)	sotigalimab/APX005M	Conventional mAb	Systemic	Neoadjuvant chemoradiotherapy combination	Esophageal/GEJ cancer	33	Yes (Pools 2/4)
Bentebibel et al., 2026 (39)	sotigalimab/APX005M	Conventional mAb	Intratumoral/local	ICI combination (pembrolizumab)	Melanoma	32	No
Osorio et al., 2025 (40)	2141-V11	Fc-engineered/Fc-optimized mAb	Intratumoral/local	Monotherapy	Basket/mixed solid tumors	12	No
Coveler et al., 2023 (14)	SEA-CD40	Fc-engineered/Fc-optimized mAb	Systemic	Mixed monotherapy/combination	Mixed solid tumors and lymphomas	67	No
Luke et al., 2021 (13)	ABBV-428	Tumor-targeted/bispecific CD40 agonist	Systemic	Monotherapy	Basket/mixed solid tumors	59	Yes (Pool 1)
Melero et al., 2026 (41)	RO7300490	Tumor-targeted/bispecific CD40 agonist	Systemic	Monotherapy	Basket/mixed solid tumors	80	Yes (Pool 1)
Tran et al., 2024 (42)	MEDI5083	Trimerized CD40L–IgG4 Fc-fusion protein	Systemic	ICI combination (durvalumab)	Basket/mixed solid tumors	38	No
Steeghs et al., 2026 (43)	MP0317	FAP×CD40 DARPin-based construct	Systemic	Monotherapy	Basket/mixed solid tumors	46	No
Lakhani et al., 2025 (44)	SL-172154	Bispecific CD47 inhibitor/CD40 agonist Fc-fusion protein	Systemic	Monotherapy	Ovarian cancer	27	Yes (Pool 1)
Drew et al., 2026 (45)	SL-172154	Bispecific CD47 inhibitor/CD40 agonist Fc-fusion protein	Systemic	Other combination (Mirvetuximab soravtansine)	Ovarian cancer	86	No

Studies are grouped by biologic format and then ordered by agent, year, and first author. Reports in the non-monoclonal-antibody category are described using their specific construct formats.

Exploratory pool indicates that a report contributed an explicit denominator and a directly reported or reproducibly derived patient-level numerator to at least one prespecified exploratory proportional-synthesis pool: Pool 1, monotherapy all-cause grade ≥3 treatment-emergent adverse events; Pool 2, combination all-cause grade ≥3 treatment-emergent adverse events; Pool 3, treatment-related grade ≥3 adverse events; and Pool 4, any-grade treatment-related adverse events.

The SEA-CD40 report included patients with solid tumors and lymphomas; a solid-tumor-specific high-grade safety denominator could not be isolated.Abbreviations: CD40L, CD40 ligand; DARPin, designed ankyrin repeat protein; Fc, fragment crystallizable; GEJ, gastroesophageal junction; ICI, immune checkpoint inhibitor; IgG4, immunoglobulin G4; mAb, monoclonal antibody; mFOLFIRINOX, modified folinic acid, fluorouracil, irinotecan, and oxaliplatin; PDAC, pancreatic ductal adenocarcinoma.

By biologic format, 19 reports evaluated conventional anti-CD40 monoclonal antibodies, two evaluated Fc-engineered or Fc-optimized antibodies, two evaluated tumor-targeted or bispecific CD40 agonists, and four evaluated non-monoclonal-antibody constructs, including designed ankyrin repeat protein–based and fusion-protein approaches. Systemic administration predominated (24 reports), whereas three reports evaluated intratumoral or local delivery. The distribution across format and route was therefore markedly uneven, with conventional systemically administered antibodies forming the largest evidence stratum.

Treatment context also varied substantially. Ten reports evaluated monotherapy, one included both monotherapy and combination cohorts, and 16 evaluated combination regimens. The principal combination partners were chemotherapy in five reports, an immune checkpoint inhibitor in five, chemotherapy plus an immune checkpoint inhibitor in one, radiotherapy or chemoradiotherapy in one, vaccine-based therapy in two, and other anticancer or immunotherapeutic combinations in two. These combination studies differed in treatment partner, sequence, disease setting, and approach to adverse-event attribution.

Tumor coverage was broad but uneven. Basket or mixed solid-tumor populations accounted for 14 reports, followed by pancreatic ductal adenocarcinoma in five, melanoma in four, ovarian cancer in two, esophageal or gastroesophageal junction cancer in one, and mesothelioma in one. No report was dedicated exclusively to non-small-cell lung cancer, although this histology was represented within several basket or mixed-tumor populations.

Seventeen of the 19 conventional-antibody reports used systemic administration, and nine provided patient-level high-grade safety data eligible for at least one exploratory pool. The systemic Fc-engineered stratum was represented only by SEA-CD40, for which a comparable solid-tumor-specific high-grade denominator could not be isolated. The other Fc-engineered report, evaluating 2141-V11, used intratumoral delivery. Neither report contributed to an exploratory pool. Consequently, quantitative comparison by antibody format or delivery route was not feasible.

### 3.3 Clinical activity and immunological/pharmacodynamic context

At least one clinical activity endpoint was reported for all 27 trial families (**Supplementary Table 6**). An identifiable objective response rate or disease control rate was reported in 22 trial families; the remaining studies primarily reported pathologic response, survival, stable disease, recurrence, or other study-specific activity outcomes. Clinical activity was presented descriptively because definitions, denominators, tumor settings, treatment regimens, and assessment methods were not sufficiently comparable for quantitative synthesis.

Trial-specific immunological or pharmacodynamic findings were reported in 26 of the 27 trial families (**Supplementary Table 7**). Reported domains included antigen-presenting-cell or dendritic-cell activation, circulating B-cell changes, T-cell activation or tumor infiltration, cytokine and chemokine responses, receptor or target occupancy, peripheral-blood pharmacodynamics, and paired tumor-biopsy or tumor-microenvironment findings. All 27 trial families were retained in **Supplementary Table 7**, including one combination trial that did not provide trial-specific immunological or pharmacodynamic findings, to document this absence without attributing findings from a separate monotherapy trial.

Pretreatment immune-related measurements were explicitly reported in four trial families, but the measured variables and analytical methods differed. Only one trial family described a temporal association between post-infusion tumor necrosis factor-α and interleukin-6 elevations and grade 1–2 cytokine-release syndrome. This relationship was reported descriptively, and no causal immune–safety association was formally established.

### 3.4 Safety-outcome reporting availability

**Figure 3:**
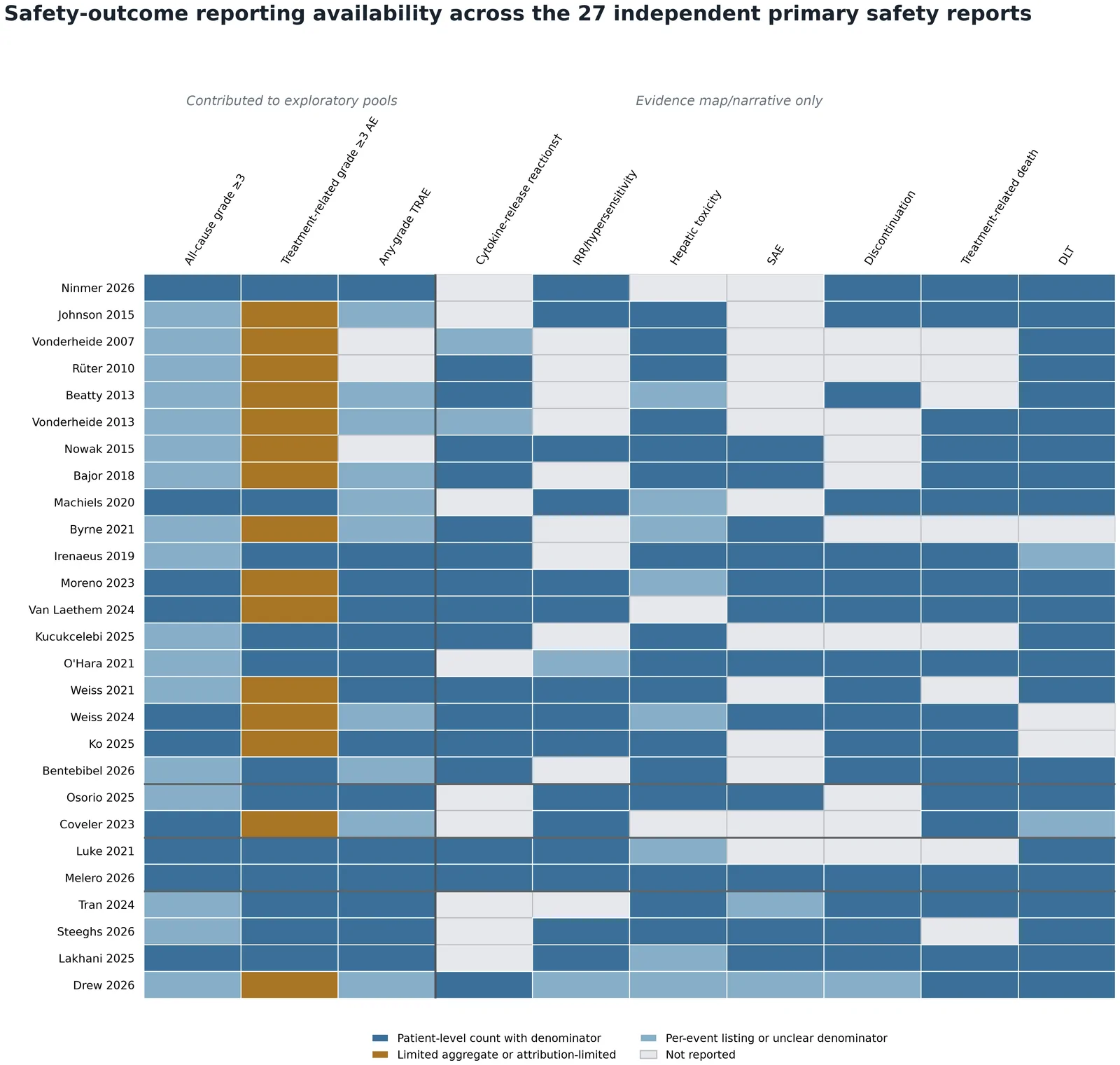
Safety-outcome reporting availability across the 27 independent primary safety reports. Cells indicate patient-level reporting, limited aggregate or attribution-limited reporting, per-event or unclear-denominator reporting, or non-reporting. Studies are ordered as in **Table 1** and grouped by biologic format. Cytokine-release reactions were not pooled because grading frameworks were heterogeneous or unspecified. Per-event listings were not converted to patient-level counts, and dose-limiting toxicity was treated separately from treatment-related grade ≥3 adverse events. AE, adverse event; CRS, cytokine-release reaction; DLT, dose-limiting toxicity; IRR, infusion-related reaction; SAE, serious adverse event; TEAE, treatment-emergent adverse event; TRAE, treatment-related adverse event.

Safety-outcome reporting varied substantially across the 27 independent primary safety reports (**Table 2**; **Figure 3**). Reporting was classified as an explicit patient-level numerator and denominator; limited aggregate or attribution-limited reporting; a per-event listing or unclear denominator; or no reporting. The availability of patient-level data did not necessarily imply eligibility for exploratory synthesis, because some reports involved non-comparable populations, treatment contexts, formats, routes, or outcome definitions. The complete study-by-outcome reporting matrix is provided in **Supplementary Table 5**.

**Table 2:** Safety-outcome reporting availability across the 27 independent primary safety reports.

Safety outcome	Reports with explicit patient-level n/N	Limited aggregate or attribution-limited reporting	Per-event listing or unclear denominator	Not reported	Exploratory synthesis
All-cause grade ≥3 TEAE	10	0	17 (per-event only)	0	Pool 1/Pool 2
Treatment-related grade ≥3 AE	12	15 (percentage only 2; all-cause only 8; treatment-related not grade-specific 5)	0	0	Pool 3
Any-grade TRAE	14	0	10 (per-event only)	3	Pool 4
Cytokine-release reactions†	16	0	2 (unclear denominator)	9	No
Infusion-related or hypersensitivity reaction	15	0	2 (unclear denominator)	10	No
Hepatic toxicity	16	0	8 (unclear denominator)	3	No
Serious adverse events	12	0	2 (unclear denominator)	13	No
Treatment discontinuation due to toxicity	16	0	1 (unclear denominator)	10	No
Treatment-related death	19	0	0	8	No
Dose-limiting toxicity	22	0	2 (unclear denominator)	3	No

Values are numbers of reports; categories are mutually exclusive and sum to 27 for each outcome.

Reports with explicit patient-level n/N provided both a patient-level numerator and denominator. Limited aggregate or attribution-limited reporting comprised percentage-only reporting, all-cause reporting without treatment-related attribution, or treatment-related reporting without grade-specific patient-level data.

Per-event listings could not be summed to patient-level any-event counts because individual patients could experience multiple events. Patient-level cytokine-release-reaction counts were available in 16 reports but were not pooled because grading frameworks were heterogeneous or unspecified.

Dose-limiting toxicity was treated as a distinct dose-finding outcome and not as a substitute for treatment-related grade ≥3 adverse events. Pool definitions are provided in **Table 3**.Abbreviations: AE, adverse event; DLT, dose-limiting toxicity; TEAE, treatment-emergent adverse event; TRAE, treatment-related adverse event.

For all-cause grade ≥3 treatment-emergent adverse events, explicit patient-level data were available in 10 reports, whereas 17 provided only per-event or preferred-term listings without a patient-level count of patients experiencing at least one grade ≥3 event. Nine of the 10 reports with patient-level data were eligible for exploratory synthesis: four monotherapy and five combination studies. SEA-CD40 was retained in the reporting map but not pooled because its mixed solid-tumor and lymphoma population did not provide a separable solid-tumor-specific denominator.

Treatment-related grade ≥3 adverse events were reported with explicit patient-level data in 12 reports. Among the remaining 15 reports, two provided percentage-only values, eight reported only all-cause grade ≥3 events without treatment-related attribution, and five reported treatment-related events without grade-specific patient-level data. The exploratory treatment-related grade ≥3 pool ultimately comprised five conventional systemically administered antibody reports, including three with directly tabulated patient-level counts and two with reproducibly derived values. Other patient-level reports did not meet the prespecified format-route or treatment-context criteria for this pool.

For any-grade treatment-related adverse events, explicit patient-level data were available in 14 reports, 10 provided only per-event or preferred-term listings, and three did not report the outcome. Per-event tables were not summed to create patient-level any-event counts because individual patients could contribute more than one adverse-event type.

Patient-level data were reported for cytokine-release reactions in 16 reports, infusion-related or hypersensitivity reactions in 15, hepatic toxicity in 16, serious adverse events in 12, treatment discontinuation due to toxicity in 16, treatment-related deaths in 19, and dose-limiting toxicities in 22. Dose-limiting toxicities were treated as a distinct dose-finding outcome and were not used as a substitute for treatment-related grade ≥3 adverse events.

Cytokine-release reactions illustrate the distinction between data availability and comparability. Although patient-level counts were available in 16 reports, grading frameworks varied or were unspecified. This outcome was therefore retained for evidence mapping and narrative synthesis rather than pooled across incompatible definitions.

The reporting assessment supported four exploratory pools: all-cause grade ≥3 treatment-emergent adverse events in monotherapy studies, all-cause grade ≥3 treatment-emergent adverse events in combination studies, treatment-related grade ≥3 adverse events, and any-grade treatment-related adverse events. These pools represented selected comparable reporting strata rather than the entire evidence base.

### 3.5 Exploratory proportional synthesis

Four prespecified outcome pools met the minimum threshold of four reports with explicit denominators and directly reported or reproducibly derived patient-level numerators (**Table 3**; **Supplementary Figure 1**).

**Table 3:** Exploratory proportional synthesis of selected safety outcomes across CD40 agonist studies.

Pool	Outcome	k	Events/N	Study-level range, %	Pooled proportion, % (95% CI)	I², %	Interpretive limitation
Pool 1	All-cause grade ≥3 TEAEs in monotherapy studies	4	133/261	38.8–61.0	50.7 (34.1–67.1)	66	Monotherapy high-grade anchor; direct patient-level counts only; mixed biologic formats; limited by small k.
Pool 2	All-cause grade ≥3 TEAEs in combination-therapy studies	5	115/200	0–78.8	50.3 (9.1–91.1)	89	Very wide confidence interval; regimen-level estimate not attributable specifically to CD40 agonism.
Pool 3	Investigator-reported treatment-related grade ≥3 AEs in the conventional/Fc-engineered systemic stratum	5	50/143	0–93.3	25.7 (1.6–88.1)	89	Very wide confidence interval; investigator-attributed outcome with unresolved contribution of combination partners; all contributing reports evaluated conventional systemic antibodies.
Pool 4	Any-grade treatment-related AEs	7	265/292	84.2–100	89.7 (80.8–94.8)	29	Consistently high any-grade frequency with limited discrimination of high-grade toxicity.

Pooled proportions were estimated using inverse-variance random-effects models of logit-transformed proportions, with restricted maximum likelihood estimation and Hartung–Knapp adjustment; a continuity correction of 0.5 was applied where required. Estimates were exploratory and hypothesis-generating rather than comparative or confirmatory. Pools 2–4 included reproducibly derived inputs; sensitivity analyses and prediction intervals are provided in **Supplementary Figure 1**. k denotes the number of contributing reports. AE, adverse event; CI, confidence interval; I², percentage of total variability attributable to between-study heterogeneity; TEAE, treatment-emergent adverse event; TRAE, treatment-related adverse event.

For all-cause grade ≥3 treatment-emergent adverse events in monotherapy studies (Pool 1), 133 of 261 patients experienced the outcome across four reports. The pooled proportion was 50.7% (95% confidence interval [CI] 34.1–67.1%; I² = 66%), and study-level proportions ranged from 38.8% to 61.0%. All inputs to this pool were directly reported patient-level counts. For all-cause grade ≥3 treatment-emergent adverse events in combination-therapy studies (Pool 2), 115 of 200 patients experienced the outcome across five reports. The pooled proportion was 50.3% (95% CI 9.1–91.1%; I² = 89%), with study-level proportions ranging from 0% to 78.8%. The wide confidence interval and substantial heterogeneity reflected marked variability across the included regimens. For investigator-reported treatment-related grade ≥3 adverse events (Pool 3), 50 of 143 patients experienced the outcome across five reports. The pooled proportion was 25.7% (95% CI 1.6–88.1%; I² = 89%), and study-level proportions ranged from 0% to 93.3%. Although this pool was defined within the conventional or Fc-engineered systemic antibody stratum, all five contributing reports evaluated conventional systemically administered antibodies; no systemic Fc-engineered report provided a comparable solid-tumor-specific denominator. For any-grade treatment-related adverse events (Pool 4), 265 of 292 patients experienced at least one event across seven reports. The pooled proportion was 89.7% (95% CI 80.8–94.8%; I² = 29%), with study-level proportions ranging from 84.2% to 100%. This pool had the narrowest confidence interval and lowest heterogeneity but captured the occurrence of any treatment-related event irrespective of severity.

Pools 2–4 incorporated values reproducibly derived from reported percentages or narrative safety statements. Sensitivity analyses excluding derived values did not materially change the overall interpretation, although the smaller number of contributing reports reduced precision. Prediction intervals and results from alternative transformations, fixed-effect analyses, generalized linear mixed models, and derived-value exclusion analyses are provided in **Supplementary Figure 1**. Given the small pool sizes, wide confidence intervals, and substantial heterogeneity in selected pools, these estimates were retained as exploratory proportional summaries rather than comparative class-level toxicity rates.

### 3.6 Study-level safety proportion patterns

**Figure 4:**
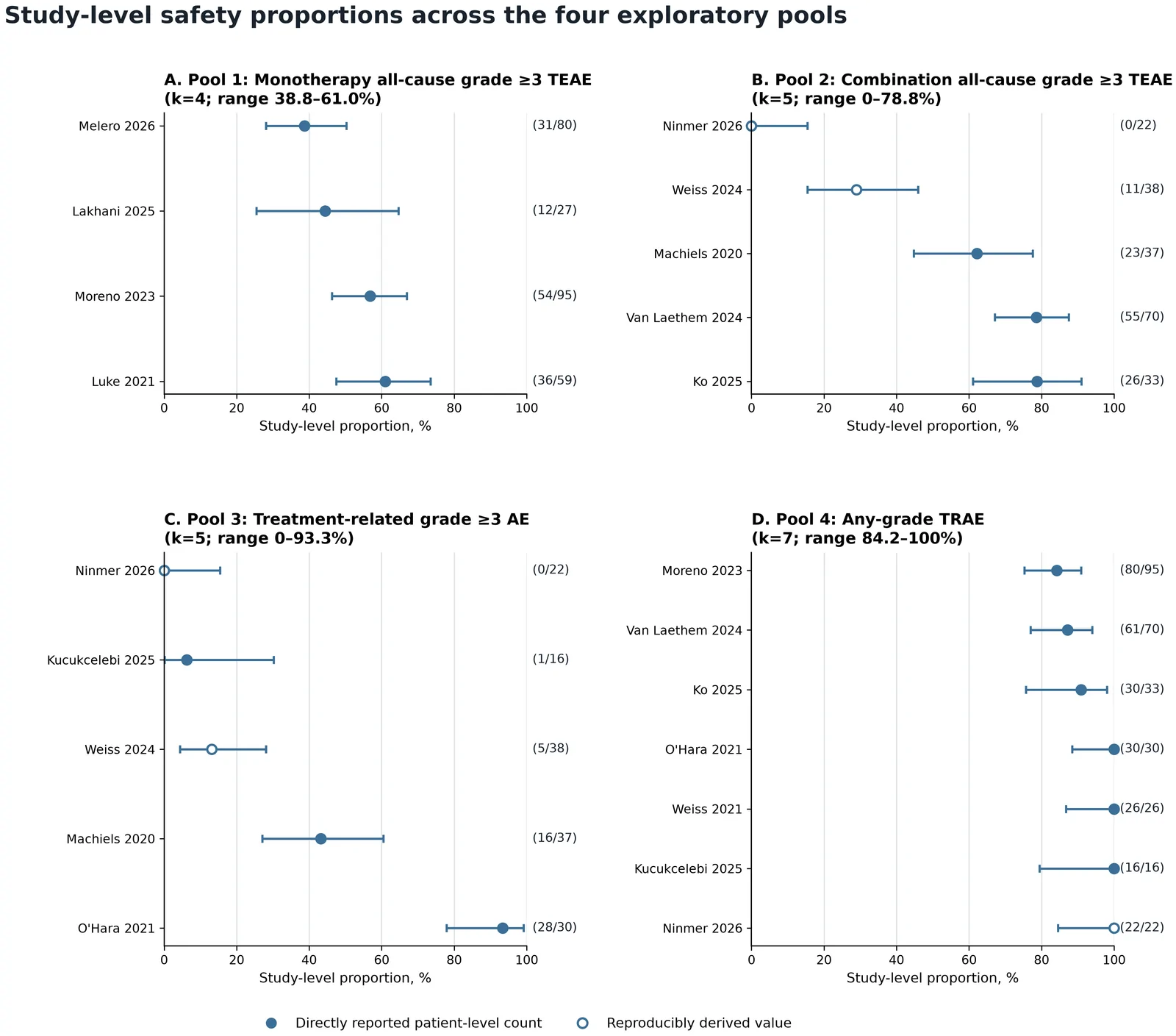
Study-level proportions across the four exploratory proportional-synthesis pools. Panels show study-level proportions with 95% Clopper–Pearson confidence intervals for **(A)** monotherapy all-cause grade ≥3 TEAEs, **(B)** combination all-cause grade ≥3 TEAEs, **(C)** treatment-related grade ≥3 AEs, and **(D)** any-grade TRAEs. Filled circles indicate directly reported counts and open circles reproducibly derived values. Pooled estimates are reported in **Table 3**, with forest plots and sensitivity analyses in **Supplementary Figure 1**. AE, adverse event; TEAE, treatment-emergent adverse event; TRAE, treatment-related adverse event.

Study-level proportions with Clopper–Pearson exact 95% confidence intervals are shown in **Figure 4**. Pooled estimates are not displayed in the figure and are reported separately in **Table 3** and **Supplementary Figure 1**.

The four monotherapy estimates contributing to Pool 1 ranged from 38.8% to 61.0% and were more closely grouped than the estimates contributing to the other high-grade pools. Combination-study estimates in Pool 2 ranged from 0% to 78.8%, while treatment-related grade ≥3 estimates in Pool 3 ranged from 0% to 93.3%. The widest study-level dispersion was therefore observed in the combination and treatment-related high-grade pools.

Any-grade treatment-related adverse-event proportions in Pool 4 ranged from 84.2% to 100%. These estimates were consistently high and showed less between-study variability than the high-grade pools. The study-level distributions illustrate the contrasting patterns underlying the four exploratory summaries: a comparatively narrower monotherapy range, marked dispersion in the combination and treatment-related high-grade strata, and consistently high any-grade treatment-related proportions.

### 3.7 Study quality and reporting limitations

Study quality was assessed using the Methodological Index for Non-Randomized Studies (MINORS) for all 27 independent primary safety reports (**Supplementary Table 4**). The median score was 12 of 16 (interquartile range 12–13; range 11–14). Linked reports within the same trial family were not assessed separately.

Inclusion of consecutive patients and unbiased endpoint assessment were inadequately reported in all 27 reports. Prospective sample-size calculation was absent or inadequate in 25 reports, and follow-up appropriate to the study aim was limited in 11. Patient-level safety reporting was incomplete in a substantial proportion of reports.

Treatment attribution was unclear in eight reports, corresponding to studies that reported all-cause grade ≥3 events without treatment-related attribution. The version of the Common Terminology Criteria for Adverse Events and the grading framework used for cytokine-release reactions were also incompletely or inconsistently reported. Overlapping safety populations required trial-family linkage to prevent duplicate denominators.

Higher MINORS scores did not necessarily indicate complete safety reporting. Among the 10 reports scoring 13 or 14, six retained at least one reporting limitation, including incomplete patient-level denominators, unclear treatment attribution, or incomplete event-grading information. Methodological quality and safety-reporting completeness therefore represented related but distinct dimensions of the evidence base.

## 4 Discussion

In this systematic review and evidence map, we characterized the clinical safety evidence for agonistic anti-CD40 antibodies and related CD40 agonist biologics in patients with solid tumors. Across 27 independent primary safety reports covering 12 agents or biologics, the evidence base was broad in nominal scope but highly uneven in depth. Most reports described early-phase studies, and the available safety evidence was concentrated in conventional systemically administered monoclonal antibodies, whereas Fc-engineered, tumor-targeted or bispecific, non-monoclonal-antibody, and intratumoral or locally delivered approaches were represented by few reports. This imbalance precluded the originally planned quantitative comparison by antibody format and delivery route.

A central finding was that the interpretability of the current safety literature was constrained as much by reporting structure as by the number of available studies. The number of patients experiencing an event and the number assessed were inconsistently reported across safety outcomes, and several reports provided only per-event listings, attribution-limited summaries, unclear denominators, or non-harmonized grading frameworks. Consequently, several clinically relevant safety domains—including cytokine-release reactions, infusion-related or hypersensitivity reactions, hepatic toxicity, serious adverse events, treatment discontinuation, treatment-related deaths, and dose-limiting toxicities—were more suitable for evidence mapping or narrative synthesis than for pooled proportional estimation.

The descriptive assessment of clinical activity and immunological or pharmacodynamic findings provided additional context while preserving the safety-focused scope of the review. Clinical activity was reported in all 27 trial families, but endpoints, populations, regimens, and assessment methods were too heterogeneous for formal cross-study comparison. Trial-specific immunological or pharmacodynamic findings were available in 26 trial families and documented pharmacodynamic activity across several domains, including antigen-presenting-cell activation, circulating B-cell changes, T-cell activation, cytokine or chemokine responses, target occupancy, and tumor microenvironment remodeling. However, pretreatment immune-related measurements were reported in only four trial families, and only one study described a temporal association between cytokine elevations and a safety event. These findings do not support comparative efficacy conclusions or causal linkage between immune activation and toxicity.

**Figure 5:**
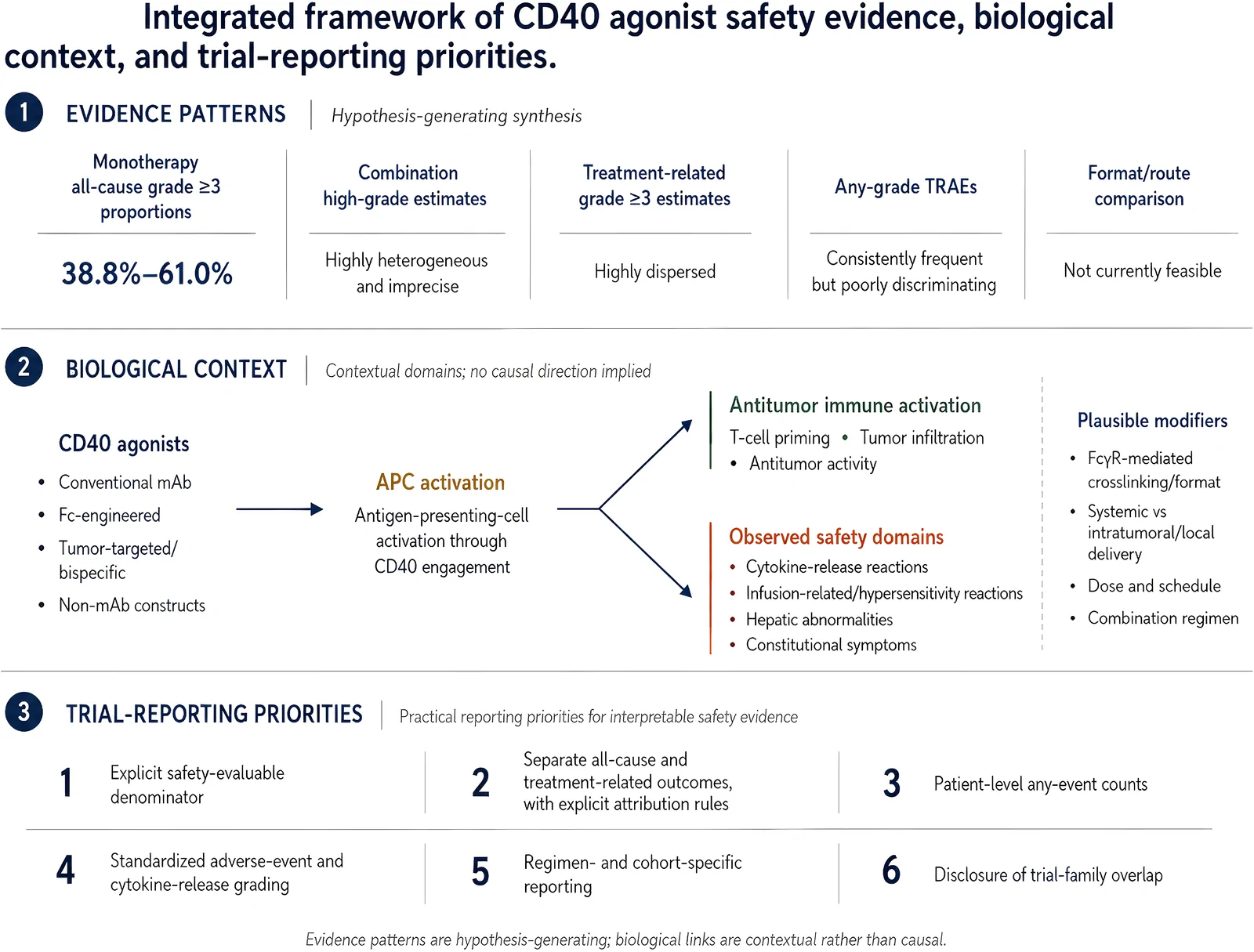
Integrated framework of CD40 agonist safety evidence, biological context, and reporting priorities. The framework summarizes the main evidence patterns, places them within the biological context of CD40-mediated antigen-presenting-cell activation, and identifies priorities for prospective safety reporting. Format, Fcγ-receptor engagement, delivery route, dose and schedule, tumor context, and combination regimen are shown as plausible modifiers rather than established determinants of toxicity. The figure is conceptual and hypothesis-generating and does not imply causal or comparative safety conclusions. APC, antigen-presenting cell; FcγR, Fc gamma receptor; mAb, monoclonal antibody; TRAE, treatment-related adverse event.

Exploratory proportional synthesis was therefore restricted to four selected reporting strata. The monotherapy all-cause grade ≥3 estimates were more closely grouped, whereas the combination and treatment-related high-grade pools were highly heterogeneous and imprecise. Any-grade treatment-related adverse events were consistently frequent but offered limited information about clinically consequential high-grade toxicity. The principal contribution of this review is therefore not a single pooled toxicity rate, but a structured account of where CD40 agonist safety evidence is available, where reporting remains incomplete, and which observations warrant prospective evaluation. **Figure 5** integrates these evidence patterns with their biological context and implications for future trial design.

The apparent breadth of the CD40 agonist field masks a highly uneven evidence base. Although 12 distinct agents or biologics have entered clinical evaluation in solid tumors, the available safety evidence is concentrated in several conventional monoclonal antibodies—principally CP-870,893/selicrelumab, sotigalimab, and mitazalimab—whereas most other agents contributed only a single early-phase report (10–12, 15). This concentration reflects the developmental stage of the field rather than a deliberate comparative design: most reports describe dose-escalation, dose-expansion, or basket cohorts in which safety reporting was primarily descriptive and not structured for cross-study comparison.

The imbalance is most consequential along the format and route dimensions that the review originally sought to compare. Conventional systemically administered antibodies formed the largest safety-reporting stratum, whereas Fc-engineered, tumor-targeted or bispecific, and non-monoclonal-antibody constructs were each represented by very few reports, and intratumoral or local delivery by only three. The single systemic Fc-engineered report, SEA-CD40, enrolled a mixed solid-tumor and lymphoma population from which a solid-tumor-specific high-grade denominator could not be isolated; it therefore contributed to the evidence map but not to any exploratory pool (14). Consequently, a direct comparison of safety by antibody format or delivery route was not feasible, even though Fc gamma receptor engagement, antibody format, and route of administration remain biologically plausible modifiers of immune activation and toxicity (40, 41, 46, 47).

Tumor coverage showed a similar pattern. Basket or mixed solid-tumor populations, pancreatic ductal adenocarcinoma, and melanoma accounted for most reports, whereas no study was dedicated exclusively to non-small cell lung cancer despite its inclusion within several mixed cohorts (11, 12, 37). The evidence base therefore nominally spans many tumor types but remains concentrated in a few settings, limiting tumor-specific safety inference. The diversity of agents, formats, routes, and tumor settings is not yet matched by the depth of comparable patient-level safety data required for quantitative comparison (9).

The four exploratory pools should be interpreted as quantitative summaries of selected reporting strata rather than as estimates of CD40 agonist class toxicity. The monotherapy all-cause grade ≥3 pool was the most internally consistent and served as the monotherapy high-grade anchor because confounding from combination partners was absent. Nevertheless, it included only four reports, involved heterogeneous agents or formats, and used an all-cause definition that did not isolate events attributable to CD40 agonism. Its study-level proportions of 38.8%–61.0% should therefore be viewed as a tentative descriptive range rather than as a definitive monotherapy toxicity rate.

The combination all-cause and treatment-related high-grade pools were both highly heterogeneous and imprecise, with confidence intervals—and prediction intervals reported in **Supplementary Figure 1** where estimable—spanning wide ranges. This dispersion should not be dismissed as statistical noise because it reflects differences in treatment regimens, tumor settings, partner therapies, dose and schedule, and attribution rules. In combination studies, the contribution of CD40 agonism could not be separated from chemotherapy, chemoradiation, vaccine-based therapy, or other multi-agent backbones. These pools are therefore best understood as regimen-level summaries, and attribution of their pooled values to CD40 agonism alone would be misleading. Furthermore, although the treatment-related grade ≥3 pool was defined within the conventional or Fc-engineered systemic stratum, all contributing reports evaluated conventional systemic antibodies; it could not test whether Fc engineering modifies high-grade toxicity.

The any-grade treatment-related pool was the only pool with a relatively narrow confidence interval and low heterogeneity, but its clinical discrimination was limited. A very high frequency of at least one any-grade treatment-related event is unsurprising in early-phase oncology studies and does not reliably distinguish agents or regimens according to clinically consequential high-grade toxicity. Across the four pools, statistical precision and clinical informativeness did not coincide: the most precise estimate was the least discriminating, whereas the estimates most closely aligned with attribution-specific high-grade toxicity were the least precise.

Four hypothesis-generating observations follow from these results. First, the comparatively narrower monotherapy range may provide a descriptive interval to be prospectively tested in future studies using standardized high-grade outcome definitions; it should not be used directly as a definitive planning parameter. Second, the marked dispersion in combination studies suggests that partner regimen and clinical context may contribute importantly to the observed variation in high-grade event rates, supporting regimen-specific reporting and stratification. Third, any-grade treatment-related events may be poorly suited as the principal endpoint for comparing the clinically important safety of different CD40 agonist formats; high-grade, attribution-specific outcomes are likely to be more informative. Fourth, whether Fc engineering, tumor targeting, or local delivery modifies high-grade toxicity remains unresolved and requires standardized prospective cohorts or direct comparative evaluation. These observations complement, but do not substitute for, the safety evidence map and reporting-availability assessment.

The pattern of safety signals observed across this evidence base can be placed in a biologically plausible context, although the available studies were not designed to attribute individual toxicities mechanistically. CD40 is expressed on antigen-presenting cells, including dendritic cells, macrophages, and B cells, and agonistic engagement is intended to mimic CD40–CD40L signaling by activating and licensing antigen-presenting cells, increasing costimulatory signaling, and promoting tumor-specific T-cell priming and infiltration (1, 2). The immune activation underlying this intended effect may also provide a plausible basis for safety domains frequently reported in these trials, including cytokine-release reactions, infusion-related or hypersensitivity reactions, hepatic laboratory abnormalities, and constitutional symptoms (10, 28). These events may reflect, at least in part, transient cytokine-mediated inflammatory responses rather than a uniform organ-specific toxicity mechanism (14, 29).

This framework also helps explain why format, route, and regimen repeatedly emerged as important dimensions of the evidence map. For many agonistic antibodies, CD40 activation is influenced by Fc gamma receptor–mediated crosslinking, making antibody format and Fc engineering biologically plausible modifiers of the intensity of immune activation and, potentially, toxicity (5, 6, 46, 48). Systemic and intratumoral or local delivery differ in the distribution of exposure, while combination with chemotherapy, chemoradiation, immune checkpoint inhibition, or vaccines introduces additional and potentially overlapping toxicities. These considerations are consistent with the heterogeneity observed in the combination and treatment-related pools, in which high-grade events could not be separated from the contributions of partner therapies.

Importantly, this mechanistic context is intended to interpret, not establish, the observed safety pattern. The included studies did not provide the controlled comparisons, standardized mechanistic endpoints, or attribution detail required to link specific toxicities to specific pathways. Sparse evidence in the Fc-engineered, bispecific, tumor-targeted, and non-monoclonal-antibody strata means that the influence of format and route on safety remains a hypothesis rather than a demonstrated effect. **Figure 5** therefore contextualizes the observed safety domains and evidence patterns without asserting causal pathways.

One of the most actionable findings concerns not only the methodological characteristics of the underlying studies but also the structure and completeness of safety reporting. MINORS scores clustered within a narrow range, consistent with the early-phase, single-arm designs that dominate the field (23). Methodological score and reporting completeness were not interchangeable: several reports with higher scores nonetheless lacked an explicit patient-level safety denominator, a clear basis for treatment attribution, the Common Terminology Criteria for Adverse Events version applied, or a specified cytokine-release grading framework. A well-conducted trial may therefore remain difficult to incorporate into quantitative safety synthesis if its results are not reported with explicit denominators, attribution, and grading information (21, 22).

Several avoidable reporting practices limited synthesis. Per-event or preferred-term adverse-event tables are informative for describing the toxicity spectrum but cannot be summed into a patient-level count because one patient may experience multiple events. Reports providing only such tables could therefore be mapped but not pooled. All-cause grade ≥3 events were frequently reported without treatment-related attribution, while dose-limiting toxicities reflect a dose-finding construct rather than the overall burden of treatment-related grade ≥3 toxicity and cannot substitute for it. Cytokine-release reactions illustrate the problem particularly clearly: patient-level counts were available in most reports, but grading frameworks varied or were unspecified, preventing synthesis across incompatible definitions (21, 22).

These findings suggest a practical safety-reporting framework for consideration in future CD40 agonist trials, consistent with broader recommendations for transparent reporting of harms (17). For every treatment arm or cohort, studies should report an explicit safety-evaluable denominator and patient-level counts for all-cause grade ≥3 treatment-emergent adverse events, treatment-related grade ≥3 adverse events, any-grade treatment-related adverse events, cytokine-release reactions, infusion-related or hypersensitivity reactions, and hepatic toxicity. Reports should specify the adverse-event grading version, cytokine-release grading framework, rules used to assign treatment relatedness, and any overlap with previously reported cohorts or registry records. Monotherapy and combination cohorts should be reported separately, and combination studies should preserve regimen- and cohort-specific safety denominators rather than combining heterogeneous treatment backbones. These priorities are summarized in **Figure 5** and are intended to improve endpoint selection, reporting consistency, and future comparability rather than to prescribe a particular trial design.

This review has several strengths. The search covered major bibliographic databases, trial registries, and conference sources, and trial-family linkage was used to prevent the same patients from contributing more than one safety denominator. All 27 independent primary safety reports were represented by peer-reviewed full-text publications; registry-only and conference-only records were used solely for trial identification, evidence mapping, or overlap clarification and did not contribute independent denominators or enter the exploratory pools. Treating safety-reporting availability as a prespecified primary output made the structure of the evidence explicit and explained why only selected outcomes could be pooled. All-cause and treatment-related events, grade ≥3 and any-grade events, and dose-limiting toxicities and treatment-related grade ≥3 toxicities were kept distinct. The evidence was also mapped across biologic format, delivery route, treatment context, and tumor setting, making gaps visible rather than obscuring them within a single pooled estimate.

This review also has important limitations, several of which reflect the underlying evidence. The included studies were predominantly early-phase, single-arm trials with small samples and heterogeneous regimens and tumor types, and patient-level safety reporting was frequently incomplete. A small number of inputs to the exploratory pools were derived from reported percentages or narrative statements rather than directly tabulated counts. Although these values were used only when the denominator was explicit and the derivation reproducible, and their exclusion did not materially change the interpretation, they introduced a degree of approximation. Sparse and non-comparable data in the Fc-engineered, bispecific, non-monoclonal-antibody, and intratumoral or local strata precluded the format- and route-based comparison originally planned. The small number of studies in each pool also precluded meaningful assessment of small-study effects.

The descriptive clinical activity and immunological or pharmacodynamic findings were highly heterogeneous and were not intended to support comparative efficacy assessment. Pretreatment immune-related measurements were reported in only a small subset of trial families, and the available evidence did not permit causal assessment of relationships between immune activation and adverse events. Finally, reliance on aggregate published reports rather than individual participant data leaves the review susceptible to selective outcome reporting and dissemination bias. Although registry and conference records did not contribute to the quantitative analyses, their limited safety detail underscores the possibility that some early-phase safety evidence remains incompletely reported or publicly available.

## 5 Conclusion

CD40 agonists have generated a heterogeneous early-phase safety evidence base spanning multiple biologic formats, delivery routes, tumor settings, and treatment regimens. Reported clinical activity and immunological or pharmacodynamic findings provide useful biological and clinical context but are too heterogeneous to support formal comparative synthesis or causal inference regarding toxicity. The current evidence supports structured safety mapping and cautious, hypothesis-generating proportional synthesis, but it does not support definitive comparative toxicity estimation across antibody format, delivery route, or treatment regimen. A major limitation lies not only in trial design and evidence sparsity but also in the heterogeneity and incompleteness of safety reporting. As newer Fc-engineered, bispecific, tumor-targeted, and locally delivered CD40 agonists advance, standardized patient-level reporting of high-grade, attribution-specific safety outcomes, with explicit denominators and grading frameworks, will be essential to enable the comparative safety assessment that the current literature cannot yet provide.

## Data Availability

The original contributions presented in the study are included in the article/**Supplementary Material**. Further inquiries can be directed to the corresponding author.
