# Supplementary material for: Agonistic anti-CD40 antibodies in patients with solid tumors: a systematic review and evidence map of clinical safety, with exploratory proportional synthesis
Source: Front Immunol. 2026 Aug 27;17:1908586. doi: 10.3389/fimmu.2026.1908586 (PMC13558018; doi:10.3389/fimmu.2026.1908586)
Supplement: Supplementary file 1 [file DataSheet1.pdf]

# Supplementary Materials

CD40 agonist systematic review and exploratory proportional syntheses

## Contents

1. Supplementary Table S1. Full search strategies for all information sources.
2. Supplementary Table S2. Characteristics of the 27 independent primary safety reports.
3. Supplementary Table S3. Trial-family linkage and overlap audit.
4. Supplementary Table S4. Per-study methodological quality (MINORS) of the 27 independent primary safety reports.
5. Supplementary Table S5. Study-level safety-outcome reporting availability across the 27 independent primary safety reports.
6. Supplementary Table S6. Reported clinical activity of included CD40 agonist trials.
7. Supplementary Table S7. Reported immunological and pharmacodynamic context of included CD40 agonist trials.
8. Supplementary Figure S1. Forest plots, prediction intervals, and sensitivity analyses for the four exploratory proportional-synthesis pools.

**Supplementary Table S1. Full search strategies for all information sources.**

All original information sources were searched on 3 June 2026. The counts below are aligned with Figure 1 and Results 3.1.

| Section | Source | Platform | Search date | Records | Notes |
|---|---|---|---|---|---|
| Database records | PubMed/ MEDLINE | NCBI PubMed | 3 June 2026 | 205 | |
| Database records | Embase | embase.com | 3 June 2026 | 561 | |
| Database records | Web of Science Core Collection | Clarivate | 3 June 2026 | 352 | |
| Database records | Scopus | Elsevier | 3 June 2026 | 612 | |
| Database records | Cochrane CENTRAL | Cochrane Library | 3 June 2026 | 35 | |
| Database records | Database records total | | 3 June 2026 | 1765 | |
| Removed before screening | Duplicate records | | | 653 | |
| Removed before screening | Ineligible publication types | | | 45 | |
| Removed before screening | Grey literature/ dissertations/ theses | | | 38 | |
| Removed before screening | Incomplete metadata | | | 30 | |
| Removed before screening | Total removed before screening | | | 766 | |
| Screening | Records screened | | | 999 | |
| Other methods | ClinicalTrials.gov | API v2 | 3 June 2026 | 85 | Unique NCT records |
| Other methods | Conference sources | ASCO/ AACR/ SITC | 3 June 2026 | 6 | |
| Other methods | Citation searching/ Google Scholar | | 3 June 2026 | 0 | |
| Other methods | Total other-method records | | 3 June 2026 | 91 | |

**1. PubMed/ MEDLINE (NCBI) — 205 records**

("CD40 agonist*"[tiab] OR "agonistic CD40"[tiab] OR "agonistic anti-CD40"[tiab] OR "anti-CD40 agonist*"[tiab] OR "CD40 agonistic antibod*"[tiab] OR sotigalimab[tiab] OR APX005M[tiab] OR selicrelumab[tiab] OR RO7009789[tiab] OR "CP-870,893"[tiab] OR "CP-870893"[tiab] OR mitazalimab[tiab] OR "ADC-1013"[tiab] OR "JNJ-64457107"[tiab] OR "SEA-CD40"[tiab] OR "CDX-1140"[tiab] OR "ChiLob 7/ 4"[tiab] OR "2141-V11"[tiab] OR RO7300490[tiab] OR "ABBV-428"[tiab] OR "SL-172154"[tiab] OR LVGN7409[tiab] OR MP0317[tiab]) AND (neoplasm*[tiab] OR cancer*[tiab] OR tumour*[tiab] OR tumor*[tiab] OR carcinoma*[tiab] OR malignan*[tiab] OR sarcoma*[tiab] OR melanoma*[tiab] OR "Neoplasms"[Mesh]) AND (patient*[tiab] OR clinical[tiab] OR trial[tiab] OR phase[tiab] OR "phase I"[tiab] OR "phase 1"[tiab] OR "phase Ib"[tiab] OR "phase II"[tiab] OR "phase 2"[tiab] OR dose-escalation[tiab] OR dose-expansion[tiab] OR safety[tiab] OR tolerability[tiab] OR toxicity[tiab] OR adverse[tiab] OR "Clinical Trial"[Publication Type]) NOT (animals[MeSH Terms] NOT humans[MeSH Terms])

**2. Embase (embase.com) — 561 records**

('CD40 agonist*':ti,ab,kw OR 'agonistic CD40':ti,ab,kw OR 'agonistic anti-CD40':ti,ab,kw OR 'anti-CD40 agonist*':ti,ab,kw OR 'CD40 agonistic antibod*':ti,ab,kw OR sotigalimab:ti,ab,kw OR APX005M:ti,ab,kw OR selicrelumab:ti,ab,kw OR RO7009789:ti,ab,kw OR 'CP-870,893':ti,ab,kw OR 'CP-870893':ti,ab,kw OR mitazalimab:ti,ab,kw OR 'ADC-1013':ti,ab,kw OR 'JNJ-64457107':ti,ab,kw OR 'SEA-CD40':ti,ab,kw OR 'CDX-1140':ti,ab,kw OR 'ChiLob 7/ 4':ti,ab,kw OR '2141-V11':ti,ab,kw OR RO7300490:ti,ab,kw OR 'ABBV-428':ti,ab,kw OR 'SL-172154':ti,ab,kw OR LVGN7409:ti,ab,kw OR MP0317:ti,ab,kw) AND ('neoplasm'/ exp OR neoplasm*:ti,ab,kw OR cancer*:ti,ab,kw OR tumour*:ti,ab,kw OR tumor*:ti,ab,kw OR carcinoma*:ti,ab,kw OR malignan*:ti,ab,kw OR sarcoma*:ti,ab,kw OR melanoma*:ti,ab,kw) AND (patient*:ti,ab,kw OR clinical:ti,ab,kw OR trial:ti,ab,kw OR phase:ti,ab,kw OR 'phase I':ti,ab,kw OR 'phase 1':ti,ab,kw OR 'phase Ib':ti,ab,kw OR 'phase II':ti,ab,kw OR 'phase 2':ti,ab,kw OR 'dose escalation':ti,ab,kw OR 'dose-escalation':ti,ab,kw OR 'dose expansion':ti,ab,kw OR 'dose-expansion':ti,ab,kw OR safety:ti,ab,kw OR tolerability:ti,ab,kw OR toxicity:ti,ab,kw OR adverse:ti,ab,kw) NOT ('animal'/ exp NOT 'human'/ exp)

**3. Web of Science Core Collection (Clarivate) — 352 records**

TS=("CD40 agonist*" OR "agonistic CD40" OR "agonistic anti-CD40" OR "anti-CD40 agonist*" OR "CD40 agonistic antibod*" OR sotigalimab OR APX005M OR selicrelumab OR RO7009789 OR "CP-870,893" OR "CP-870893" OR mitazalimab OR "ADC-1013" OR "JNJ-64457107" OR "SEA-CD40" OR "CDX-1140" OR "ChiLob 7/ 4" OR "2141-V11" OR RO7300490 OR "ABBV-428" OR "SL-172154" OR LVGN7409 OR MP0317) AND TS=(neoplasm* OR cancer* OR tumour* OR tumor* OR carcinoma* OR malignan* OR sarcoma* OR melanoma*) AND TS=(patient* OR clinical OR trial OR phase OR "phase I" OR "phase 1" OR "phase Ib" OR "phase II" OR "phase 2" OR "dose escalation" OR "dose-escalation" OR "dose expansion" OR "dose-expansion" OR safety OR tolerability OR toxicity OR adverse)

**4. Scopus (Elsevier) — 612 records**

TITLE-ABS-KEY("CD40 agonist*" OR "agonistic CD40" OR "agonistic anti-CD40" OR "anti-CD40 agonist*" OR "CD40 agonistic antibod*" OR sotigalimab OR APX005M OR selicrelumab OR RO7009789 OR "CP-870,893" OR "CP-870893" OR mitazalimab OR "ADC-1013" OR "JNJ-64457107" OR "SEA-CD40" OR "CDX-1140" OR "ChiLob 7/ 4" OR "2141-V11" OR RO7300490 OR "ABBV-428" OR "SL-172154" OR LVGN7409 OR MP0317) AND TITLE-ABS-KEY(neoplasm* OR cancer* OR tumour* OR tumor* OR carcinoma* OR malignan* OR sarcoma* OR melanoma*) AND TITLE-ABS-KEY(patient* OR clinical OR trial OR phase OR "phase I" OR "phase 1" OR "phase Ib" OR "phase II" OR "phase 2" OR "dose escalation" OR "dose-escalation" OR "dose expansion" OR "dose-expansion" OR safety OR tolerability OR toxicity OR adverse)

**5. Cochrane CENTRAL (Cochrane Library, Advanced Search; field: Title/ Abstract/ Keyword) — 35 records**

("CD40 agonist" OR "CD40 agonists" OR "agonistic CD40" OR "agonistic anti-CD40" OR "anti-CD40 agonist" OR "anti-CD40 agonists" OR "CD40 agonistic antibody" OR "CD40 agonistic antibodies" OR sotigalimab OR APX005M OR selicrelumab OR RO7009789 OR "CP-870,893" OR "CP-870893" OR mitazalimab OR "ADC-1013" OR "JNJ-64457107" OR "SEA-CD40" OR "CDX-1140" OR "ChiLob 7/ 4" OR "2141-V11" OR RO7300490

| |
|---|
| OR "ABBV-428" OR "SL-172154" OR LVGN7409 OR MP0317) AND (cancer OR neoplasm OR neoplasms OR tumor OR tumors OR tumour OR tumours OR carcinoma OR carcinomas OR malignancy OR malignancies OR sarcoma OR sarcomas OR melanoma OR melanomas) |

*CENTRAL was searched within the Trials (CENTRAL) records. 35 records were identified; all were excluded at title/ abstract screening, and none were carried forward.*

### 6. ClinicalTrials.gov (API v2) — 85 unique NCT records

Each agent name was queried individually through the API endpoint using query.term as a broad all-field search without a tumor-type restriction; solid- versus hematologic-tumor classification and eligibility were applied after retrieval.

https:/ / clinicaltrials.gov/ api/ v2/ studies?query.term=<agent name>&format=json&pageSize=100&countTotal=true

Agent names queried: CP-870,893 | CP-870893 | selicrelumab | RO7009789 | sotigalimab | APX005M |mitazalimab | ADC-1013 | JNJ-64457107 | SEA-CD40 | CDX-1140 | 2141-V11 |LVGN7409 | ChiLob 7/ 4 | ABBV-428 | SL-172154 | RO7300490 | MP0317 |dacetuzumab | SGN-40

A total of 128 term-level hits were retrieved, corresponding to 85 unique NCT records after removing alias and agent overlap. Records identifying agents evaluated only in hematological malignancies (dacetuzumab/ SGN-40) were removed during screening.

### 7. Additional sources

Conference sources (ASCO, AACR, and SITC) were checked to support trial identification and linkage. Citation searching and Google Scholar did not add any records to the locked screening count.

### Targeted alias verification undertaken during revision

- This targeted alias verification was not a rerun of the full search.
- The original eligibility criteria and the original search cutoff of 3 June 2026 were retained.
- RG7876/ RG-7876 is a development code for selicrelumab and was covered by the original selicrelumab, CP-870,893, and RO7009789 concepts.
- Targeted verification of giloralimab/ ABBV-927 identified no additional eligible, independent, result-bearing primary safety report available by 3 June 2026.
- The verification did not change the 27-report evidence base or membership of any exploratory pool.

**Supplementary Table S2. Characteristics of the 27 independent primary safety reports.**

| Study | Trial registration | Phase/ design | Agent | Alternative names | Biologic format | Route | Treatment context | Combination partner | Tumor setting | Treatment setting | Safety-evaluable N | Exploratory pool(s) |
|---|---|---|---|---|---|---|---|---|---|---|---|---|
| **Ninmer et al., 2026** | NCT04364230 | Phase I/ II, open-label, dose-escalation | CDX-1140 | — | Conventional mAb | Systemic (subcutaneous/ intradermal administration) | Vaccine-based combination (melanoma helper peptide vaccine) | Melanoma helper peptide vaccine | Melanoma | adjuvant | 22 | Pools 2, 3, and 4 |
| **Johnson et al., 2015** | NCT01561911 | Phase I, open-label, single-arm, dose-escalation | ChiLob 7/ 4 | ChiLob7/ 4 | Conventional mAb | Systemic | Monotherapy | — | Basket/ mixed solid tumors | advanced | 28 | None |
| **Vonderheide et al., 2007** | NR | Phase I, open-label, dose-escalation | CP-870,893 / selicrelumab | — | Conventional mAb | Systemic | Monotherapy | — | Basket/ mixed solid tumors | advanced | 29 | None |
| **Rüter et al., 2010** | NR | Phase I, open-label, single-arm, dose-escalation | CP-870,893 / selicrelumab | — | Conventional mAb | Systemic | Monotherapy | — | Basket/ mixed solid tumors | advanced | 27 | None |
| **Beatty et al., 2013** | NCT00711191 | Phase I, open-label, single-arm, dose-escalation | CP-870,893 / selicrelumab | — | Conventional mAb | Systemic | Chemotherapy combination (gemcitabine) | gemcitabine | PDAC | advanced/ metastatic (palliative) | 21 | None |
| **Vonderheide et al., 2013** | NCT00607048 | Phase I, open-label, dose-escalation | CP-870,893 / selicrelumab | — | Conventional mAb | Systemic | Chemotherapy combination (carboplatin) | carboplatin | Basket/ mixed solid tumors | advanced | 32 | None |
| **Nowak et al., 2015** | NR | Phase Ib, dose-escalation | CP-870,893 / selicrelumab | — | Conventional mAb | Systemic | Chemotherapy combination (cisplatin + pemetrexed) | cisplatin + pemetrexed | Mesothelioma | advanced | 15 | None |
| **Bajor et al., 2018** | NCT01103635 | Phase I, single-arm, dose-escalation | CP-870,893 / selicrelumab | CP-870,893 (with tremelimumab) | Conventional mAb | Systemic | ICI combination (tremelimumab) | tremelimumab | Melanoma | advanced/ metastatic | 24 | None |
| **Machiels et al., 2020** | NCT02760797 | Phase Ib, open-label, dose-escalation | CP-870,893 / selicrelumab | RO7009789 | Conventional mAb | Systemic | Other combination (emactuzumab) | emactuzumab | Basket/ mixed solid tumors | advanced | 37 | Pools 2 and 3 |
| **Byrne et al., 2021** | NCT02588443 | Phase I, open-label | CP-870,893 / selicrelumab | RO7009789 / RG7876 / CP-870,893 | Conventional mAb | Systemic | Chemotherapy combination (gemcitabine + nab-paclitaxel) | gemcitabine + nab-paclitaxel | PDAC | neoadjuvant and adjuvant (resectable) | 16 | None |
| **Irenaeus et** | NCT023797 | Phase I, open- | mitazalima | ADC-1013 | Conventional | Intratumoral/ local | Monotherapy | — | Basket/ mixed | advanced | 23 | None |

| Study | Trial registration | Phase/ design | Agent | Alternative names | Biologic format | Route | Treatment context | Combination partner | Tumor setting | Treatment setting | Safety-evaluable N | Exploratory pool(s) |
|---|---|---|---|---|---|---|---|---|---|---|---|---|
| al., 2019 | 41 | label, dose-escalation | b / ADC-1013 | (later mitazalimab) | mAb | | | | solid tumors | | | |
| Moreno et al., 2023 | NCT02829099 | Phase I, open-label, dose-escalation | mitazalimab / ADC-1013 | JNJ-64457107; ADC-1013 | Conventional mAb | Systemic | Monotherapy | — | Basket/ mixed solid tumors | advanced | 95 | Pools 1 and 4 |
| Van Laethem et al., 2024 | NCT04888312 | Phase Ib/ II, open-label, single-arm, dose-escalation | mitazalimab / ADC-1013 | ADC-1013; JNJ-64457107 | Conventional mAb | Systemic | Chemotherapy combination (mFOLFIRINOX) | mFOLFIRINOX | PDAC | advanced/ metastatic (palliative) | 70 | Pools 2 and 4 |
| Kucukcelebi et al., 2025 | NCT05650918 | Phase I, open-label, dose-escalation | mitazalimab / ADC-1013 | ADC-1013 | Conventional mAb | Systemic | Vaccine-based combination (MesoPher autologous dendritic cell vaccine) | MesoPher autologous dendritic cell vaccine | PDAC | advanced/ metastatic | 16 | Pools 3 and 4 |
| O'Hara et al., 2021 | NCT03214250 | Phase Ib, open-label | sotigalimab / APX005M | APX005M; sotigalimab | Conventional mAb | Systemic | Chemotherapy with or without ICI combination (gemcitabine plus nab-paclitaxel, with or without nivolumab) | Gemcitabine plus nab-paclitaxel, with or without nivolumab | PDAC | advanced/ metastatic (palliative) | 30 | Pools 3 and 4 |
| Weiss et al., 2021 | NCT03502330 | Phase I, dose-escalation | sotigalimab / APX005M | APX005M | Conventional mAb | Systemic | Cabiralizumab-based combination, with or without nivolumab | Cabiralizumab, with or without nivolumab | Basket/ mixed solid tumors | advanced | 26 | Pools 4 |
| Weiss et al., 2024 | NCT04337931 | Phase II, open-label, single-arm, dose-escalation | sotigalimab / APX005M | APX005M | Conventional mAb | Systemic | ICI combination (nivolumab) | nivolumab | Melanoma | advanced/ metastatic (palliative) | 38 | Pools 2 and 3 |
| Ko et al., 2025 | NCT03165994 | Phase II, open-label, single-arm | sotigalimab / APX005M | APX005M | Conventional mAb | Systemic | Neoadjuvant chemoradiotherapy combination | Concurrent chemoradiation: weekly carboplatin | Esophageal/ GEJ cancer | neoadjuvant | 33 | Pools 2 and 4 |
| Bentebibel et al., 2026 | NCT02706353 | Phase I/ II, open-label, dose-escalation | sotigalimab / APX005M | APX005M | Conventional mAb | Intratumoral/ local | ICI combination (pembrolizumab) | pembrolizumab | Melanoma | advanced | 32 | None |
| Osorio et al., 2025 | NCT04059588 | Phase I, open-label, dose-escalation | 2141-V11 | Fc-engineered IgG1 anti-CD40 agonistic antibody (IgG1 variant of clone CP-870,893) | Fc-engineered/ Fc-optimized mAb | Intratumoral/ local | Monotherapy | — | Basket/ mixed solid tumors | advanced/ metastatic | 12 | None |
| Coveler et al., 2023 | NCT02376699 | Phase I, open-label, dose-escalation | SEA-CD40 | non-fucosylated anti-CD40 mAb derived from | Fc-engineered/ Fc-optimized mAb | Systemic | Mixed monotherapy/ combination | — | Mixed solid tumors and lymphomas | advanced/ metastatic (palliative) | 67 | None |

| Study | Trial registration | Phase/ design | Agent | Alternative names | Biologic format | Route | Treatment context | Combination partner | Tumor setting | Treatment setting | Safety-evaluable N | Exploratory pool(s) |
|---|---|---|---|---|---|---|---|---|---|---|---|---|
| | | | | dacetuzumab (SGN-40) | | | | | | | | |
| **Luke et al., 2021** | NCT02955251 | Phase I, open-label, dose-escalation | ABBV-428 | — | Tumor-targeted/ bispecific CD40 agonist | Systemic | Monotherapy | — | Basket/ mixed solid tumors | advanced | 59 | Pools 1 |
| **Melero et al., 2026** | NCT04857138 | Phase I, open-label, single-arm, dose-escalation | RO7300490 | FAP-CD40; FAP x CD40 bispecific CD40 agonist | Tumor-targeted/ bispecific CD40 agonist | Systemic | Monotherapy | — | Basket/ mixed solid tumors | advanced/ metastatic | 80 | Pools 1 |
| **Tran et al., 2024** | NCT03089645 | Phase I, open-label, dose-escalation | MEDI5083 | MEDI5083 (CD40L-Fc fusion protein; trimerized CD40L-IgG4P Fc fusion) | Trimerized CD40L–IgG4 Fc-fusion protein | Systemic | ICI combination (durvalumab) | durvalumab | Basket/ mixed solid tumors | advanced | 38 | None |
| **Steeghs et al., 2026** | NCT05098405 | Phase I, open-label, dose-escalation | MP0317 | FAP x CD40 DARPin | FAP×CD40 DARPin-based construct | Systemic | Monotherapy | — | Basket/ mixed solid tumors | advanced | 46 | None |
| **Lakhani et al., 2025** | NCT04406623 | Phase I, open-label, single-arm, dose-escalation | SL-172154 | SIRPalpha-Fc-CD40L (SIRPa-Fc-CD40L); SL03-OHD-101 | Bispecific CD47 inhibitor/ CD40 agonist Fc-fusion protein | Systemic | Monotherapy | — | Ovarian cancer | advanced/ metastatic (palliative) | 27 | Pools 1 |
| **Drew et al., 2026** | NCT05483933 | Phase Ib, open-label, dose-escalation | SL-172154 | SIRPalpha-Fc-CD40L fusion protein; bispecific CD47 inhibitor and CD40 agonist Fc-fusion protein | Bispecific CD47 inhibitor/ CD40 agonist Fc-fusion protein | Systemic | Other combination (Mirvetuximab soravtansine) | Mirvetuximab soravtansine | Ovarian cancer | advanced/ palliative | 86 | None |

**Table notes**

1. Exploratory pool(s) indicate that the report contributed an explicit denominator and a directly reported or reproducibly derived patient-level numerator to one or more prespecified proportional-synthesis pools.
2. Pool 1, monotherapy all-cause grade ≥3 TEAE; Pool 2, combination all-cause grade ≥3 TEAE; Pool 3, treatment-related grade ≥3 AE; Pool 4, any-grade TRAE.

**Supplementary Table S3. Trial-family linkage and overlap audit.**

**Panel A. Linked or overlapping full-text reports**

| Trial family | Trial registration | Report | Report type/ role | Population relationship | Denominator handling | Use in this review |
|---|---|---|---|---|---|---|
| PRINCE | NCT03214250 | O'Hara et al., 2021 | Independent primary safety report | Phase Ib safety population within the PRINCE trial family | Primary non-overlapping denominator | Safety evidence and trial-family anchor |
| PRINCE | NCT03214250 | Padrón et al., 2022 | Linked phase II/ translational report | Overlapping PRINCE participants; translational and phase II extension | Not counted as an additional denominator | Clinical/ immune context and trial linkage |
| Neoadjuvant sotigalimab, esophageal/ GEJ | NCT03165994 | Ko et al., 2025 | Independent primary safety report | Full clinical trial report | Primary non-overlapping denominator | Safety evidence and trial-family anchor |
| Neoadjuvant sotigalimab, esophageal/ GEJ | NCT03165994 | Soto et al., 2024 | Linked translational report | Linked six-patient translational subset | Not counted as an additional denominator | Translational context only |
| CP-870,893 + gemcitabine, PDAC | NCT00711191 | Beatty et al., 2013 | Independent primary safety report | Full clinical trial report | Primary non-overlapping denominator | Safety evidence and trial-family anchor |
| CP-870,893 + gemcitabine, PDAC | NCT00711191 | Wattenberg et al., 2021 | Linked translational/ follow-up report | Same trial family; follow-up immune analysis | Not counted as an additional denominator | Translational context only |
| CP-870,893 + chemotherapy, mesothelioma | NR | Nowak et al., 2015 | Independent primary safety report | Full clinical trial report | Primary non-overlapping denominator | Safety evidence and trial-family anchor |
| CP-870,893 + chemotherapy, mesothelioma | NR | McDonnell et al., 2017 | Linked immunomonitoring report | Same treatment cohort with serial immune monitoring | Not counted as an additional denominator | Immunomonitoring context only |
| OPTIMIZE-1 | NCT04888312 | Van Laethem et al., 2024 | Independent primary safety report | Primary OPTIMIZE-1 clinical report | Primary non-overlapping denominator | Safety evidence and trial-family anchor |
| OPTIMIZE-1 | NCT04888312 | Van Laethem et al., 2025 | Linked biomarker report | Updated biomarker analysis of the same OPTIMIZE-1 trial | Not counted as an additional denominator | Biomarker context only |
| Neoadjuvant selicrelumab, PDAC | NCT02588443 | Byrne et al., 2021 | Independent primary safety report | Full clinical trial report | Primary non-overlapping denominator | Safety evidence and trial-family anchor |
| Neoadjuvant selicrelumab, PDAC | NCT02588443 | Blise et al., 2024 | Linked spatial/ translational report | Same trial family; spatial/ translational analysis | Not counted as an additional denominator | Translational context only |

**Panel B. Registry-/ conference-only records retained for mapping/ linkage**

| Trial/ agent | Registration or conference identifier | Record type | Purpose in review | Independent denominator used? | Entered exploratory pool? | Linked primary report, if applicable |
|---|---|---|---|---|---|---|
| sotigalimab/ APX005M | NCT02600949 | Registry-only | Trial identification and evidence mapping | No | No | None |
| SL-172154 | NCT04502888 | Registry-only | Trial identification and evidence mapping | No | No | None |
| LVGN7409 | NCT05075993 | Registry-only | Trial identification and evidence mapping | No | No | None |
| CDX-1140 | NCT05349890 | Registry-only | Trial identification and evidence mapping | No | No | None |
| LVGN7409 | NCT06159621 | Registry-only | Trial identification and evidence mapping | No | No | None |
| 2141-V11 | NCT06347705 | Registry-only | Trial identification and evidence mapping | No | No | None |
| CDX-1140 | NCT06980038 | Registry-only | Trial identification and evidence mapping | No | No | None |
| MP0317 | NCT07036380 | Registry-only | Trial identification and evidence mapping | No | No | None |

| Trial/ agent | Registration or conference identifier | Record type | Purpose in review | Independent denominator used? | Entered exploratory pool? | Linked primary report, if applicable |
|---|---|---|---|---|---|---|
| mitazalimab/ ADC-1013 | NCT07199764 | Registry-only | Trial identification and evidence mapping | No | No | None |
| mitazalimab/ ADC-1013 | NCT07319195 | Registry-only | Trial identification and evidence mapping | No | No | None |
| mitazalimab/ ADC-1013 | NCT07437287 | Registry-only | Trial identification and evidence mapping | No | No | None |
| CDX-1140 | NCT03329950; AACR 2019 LB-194 ; SITC 2018 (digitalcommons.providence.org/ sitc2018/ 8) | Conference-only | Evidence mapping and trial-family linkage/ overlap clarification | No | No | None identified by the search cutoff |
| SEA-CD40 | NCT02376699; JCO suppl (multiple ASCO years) | Conference-only | Evidence mapping and trial-family linkage/ overlap clarification | No | No | Coveler et al., 2023 |

**Table notes**

1. Each trial family contributed no more than one non-overlapping denominator for each outcome.
2. Linked reports were not counted as additional independent safety reports.
3. Registry- and conference-only records were used only for trial identification, evidence mapping, or overlap clarification and did not enter any exploratory pool.
4. The remaining 21 trial families had no linked full-text report retained in Panel A.

**Supplementary Table S4. Per-study methodological quality (MINORS) of the 27 independent primary safety reports.**

| Study | Clearly stated aim | Consecutive patients | Prospective data collection | Appropriate endpoints | Unbiased endpoint assessment | Appropriate follow-up | Loss to follow-up <5% | Prospective sample-size calculation | Total/ 16 |
|---|---|---|---|---|---|---|---|---|---|
| Ninmer et al., 2026 | 2 | 1 | 2 | 2 | 1 | 2 | 2 | 0 | 12 |
| Johnson et al., 2015 | 2 | 1 | 2 | 2 | 1 | 1 | 1 | 1 | 11 |
| Vonderheide et al., 2007 | 2 | 1 | 2 | 2 | 1 | 2 | 2 | 1 | 13 |
| Rüter et al., 2010 | 2 | 1 | 2 | 2 | 1 | 1 | 2 | 1 | 12 |
| Beatty et al., 2013 | 2 | 1 | 2 | 2 | 1 | 1 | 2 | 1 | 12 |
| Vonderheide et al., 2013 | 2 | 1 | 2 | 2 | 1 | 1 | 2 | 1 | 12 |
| Nowak et al., 2015 | 2 | 1 | 2 | 2 | 1 | 2 | 1 | 1 | 12 |
| Bajor et al., 2018 | 2 | 1 | 2 | 2 | 1 | 2 | 2 | 1 | 13 |
| Machiels et al., 2020 | 2 | 1 | 2 | 2 | 1 | 1 | 2 | 1 | 12 |
| Byrne et al., 2021 | 2 | 1 | 2 | 2 | 1 | 2 | 2 | 0 | 12 |
| Irenaeus et al., 2019 | 2 | 1 | 2 | 2 | 1 | 2 | 2 | 1 | 13 |
| Moreno et al., 2023 | 2 | 1 | 2 | 2 | 1 | 2 | 2 | 1 | 13 |
| Van Laethem et al., 2024 | 2 | 1 | 2 | 2 | 1 | 2 | 2 | 2 | 14 |
| Kucukcelebi et al., 2025 | 2 | 1 | 2 | 2 | 1 | 2 | 1 | 1 | 12 |
| O'Hara et al., 2021 | 2 | 1 | 2 | 2 | 1 | 2 | 2 | 1 | 13 |
| Weiss et al., 2021 | 2 | 1 | 2 | 2 | 1 | 2 | 2 | 1 | 13 |
| Weiss et al., 2024 | 2 | 1 | 2 | 2 | 1 | 2 | 2 | 2 | 14 |
| Ko et al., 2025 | 2 | 1 | 2 | 2 | 1 | 2 | 2 | 0 | 12 |
| Bentebibel et al., 2026 | 2 | 1 | 2 | 2 | 1 | 1 | 2 | 1 | 12 |
| Osorio et al., 2025 | 2 | 1 | 2 | 2 | 1 | 1 | 2 | 1 | 12 |
| Coveler et al., 2023 | 2 | 1 | 2 | 2 | 1 | 1 | 2 | 1 | 12 |
| Luke et al., 2021 | 2 | 1 | 2 | 2 | 1 | 1 | 2 | 1 | 12 |
| Melero et al., 2026 | 2 | 1 | 2 | 2 | 1 | 1 | 2 | 1 | 12 |
| Tran et al., 2024 | 2 | 1 | 2 | 2 | 1 | 1 | 2 | 1 | 12 |
| Steeghs et al., 2026 | 2 | 1 | 2 | 2 | 1 | 2 | 2 | 1 | 13 |
| Lakhani et al., 2025 | 2 | 1 | 2 | 2 | 1 | 2 | 2 | 1 | 13 |
| Drew et al., 2026 | 2 | 1 | 2 | 2 | 1 | 2 | 1 | 1 | 12 |

**Table notes**

1. Each item was scored 0 (not reported), 1 (reported but inadequate), or 2 (reported and adequate); maximum total = 16. Linked reports were not assessed separately. The Cochrane Risk of Bias 2 tool was not applied because no randomized between-arm effect estimates were synthesized.

**Supplementary Table S5. Study-level safety-outcome reporting availability across the 27 independent primary safety reports.**

| Study | Biologic format | All-cause grade ≥3 TEAE | Treatment-related grade ≥3 AE | Any-grade TRAE | Cytokine-release reactions | Infusion-related or hypersensitivity reactions | Hepatic toxicity | Serious adverse events | Treatment discontinuation due to toxicity | Treatment-related death | Dose-limiting toxicity |
|---|---|---|---|---|---|---|---|---|---|---|---|
| **Ninmer et al., 2026** | Conventional mAb | P | P | P | NR | P | NR | NR | P | P | P |
| **Johnson et al., 2015** | Conventional mAb | E | L | E | NR | P | P | NR | P | P | P |
| **Vonderheide et al., 2007** | Conventional mAb | E | L | NR | E | NR | P | NR | NR | NR | P |
| **Rüter et al., 2010** | Conventional mAb | E | L | NR | P | NR | P | NR | NR | NR | P |
| **Beatty et al., 2013** | Conventional mAb | E | L | E | P | NR | E | NR | P | NR | P |
| **Vonderheide et al., 2013** | Conventional mAb | E | L | E | E | NR | P | NR | NR | P | P |
| **Nowak et al., 2015** | Conventional mAb | E | L | NR | P | P | P | P | NR | P | P |
| **Bajor et al., 2018** | Conventional mAb | E | L | E | P | NR | P | P | NR | P | P |
| **Machiels et al., 2020** | Conventional mAb | P | P | E | NR | P | E | NR | P | P | P |
| **Byrne et al., 2021** | Conventional mAb | E | L | E | P | NR | E | P | NR | NR | NR |
| **Irenaeus et al., 2019** | Conventional mAb | E | P | P | P | NR | P | P | P | P | E |
| **Moreno et al., 2023** | Conventional mAb | P | L | P | P | P | E | P | P | P | P |
| **Van Laethem et al., 2024** | Conventional mAb | P | L | P | P | P | NR | P | P | P | P |
| **Kucukcelebi et al., 2025** | Conventional mAb | E | P | P | P | NR | P | NR | NR | NR | P |
| **O'Hara et al., 2021** | Conventional mAb | E | P | P | NR | E | P | P | P | P | P |
| **Weiss et al., 2021** | Conventional mAb | E | L | P | P | P | P | NR | P | NR | P |
| **Weiss et al., 2024** | Conventional mAb | P | L | E | P | P | E | P | P | P | NR |
| **Ko et al., 2025** | Conventional mAb | P | L | P | P | P | P | NR | P | P | NR |
| **Bentebibel et al., 2026** | Conventional mAb | E | P | E | P | NR | P | NR | P | P | P |
| **Osorio et al., 2025** | Fc-engineered/ Fc-optimized mAb | E | P | P | NR | P | P | P | NR | P | P |
| **Coveler et al., 2023** | Fc-engineered/ Fc-optimized mAb | P | L | E | NR | P | NR | NR | NR | P | E |
| **Luke et al., 2021** | Tumor-targeted/ bispecific CD40 agonist | P | P | P | P | P | E | NR | NR | NR | P |

| | | | | | | | | | | | |
|---|---|---|---|---|---|---|---|---|---|---|---|
| **Melero et al., 2026** | Tumor-targeted/ bispecific CD40 agonist | P | P | P | P | P | P | P | P | P | P |
| **Tran et al., 2024** | Trimerized CD40L–IgG4 Fc-fusion protein | E | P | P | NR | NR | P | E | P | P | P |
| **Steeghs et al., 2026** | FAP×CD40 DARPin-based construct | E | P | P | NR | P | P | P | P | NR | P |
| **Lakhani et al., 2025** | Bispecific CD47 inhibitor/ CD40 agonist Fc-fusion protein | P | P | P | NR | P | E | P | P | P | P |
| **Drew et al., 2026** | Bispecific CD47 inhibitor/ CD40 agonist Fc-fusion protein | E | L | E | P | E | E | E | E | P | P |

**Table notes**

1. P, patient-level count with denominator; L, limited aggregate or attribution-limited reporting; E, per-event listing or unclear denominator; NR, not reported.
2. Per-event listings cannot be summed to a patient-level any-event count because individual patients may experience multiple events.
3. Dose-limiting toxicity is an independent dose-finding outcome and does not substitute for treatment-related grade ≥3 adverse events.
4. Patient-level cytokine-release-reaction counts were not pooled because grading frameworks were heterogeneous or unspecified.

**Supplementary Table S6. Reported clinical activity of included CD40 agonist trials.**

| Study | Agent | Format and route | Tumor setting | Treatment context | Phase | Clinical activity endpoint(s) | Result as reported | Primary/ linked report(s) | Notes |
|---|---|---|---|---|---|---|---|---|---|
| **Ninmer et al., 2026** | CDX-1140 | Conventional mAb; Systemic (subcutaneous/ intradermal administration) | High-risk melanoma (cutaneous, mucosal, or ocular primary sites); 11/ 22 (50%) ocular melanoma | Vaccine-based combination (melanoma helper peptide vaccine) | Phase I/ II | ORR/ DCR: NA; DFS; OS; recurrence | All participants were clinically free of disease before enrollment. Two- and three-year DFS were 59% (95% CI 41–84) and 53% (95% CI 36–80), respectively; two- and three-year OS were 91% (95% CI 80–100) and 73% (95% CI 46–100). Disease recurrence occurred in 10/ 22 (45%), and 3/ 22 (14%) died during follow-up, all from disease. | Ninmer et al., 2026 | Adjuvant setting; ORR and DCR are not applicable. |
| **Johnson et al., 2015** | ChiLob 7/ 4 | Conventional mAb; Systemic | CD40-expressing advanced solid tumors (mesothelioma, melanoma, NSCLC, colorectal, cervix, laryngeal squamous, esophageal/ pancreatic adenocarcinoma, thymic carcinoma) and diffuse large B-cell lymphoma | Monotherapy | Phase I | Stable disease; time to progression; OS | Twenty-nine treatment courses were administered to 28 patients. Stable disease at the week-8 final study visit occurred in 15 treatment courses; the median time to progression for those courses was six months. | Johnson et al., 2015 | The denominator is treatment courses, not 29 independent patients; no patient-level DCR is presented. |
| **Vonderheide et al., 2007** | CP-870,893 / selicrelumab | Conventional mAb; Systemic | advanced solid tumors (melanoma, NSCLC, sarcoma, cholangiocarcinoma, breast, thyroid, unknown primary, mesothelioma) | Monotherapy | Phase I | Objective response; stable disease | Four patients had objective partial responses at day-43 restaging (14% of all patients; 27% of patients with melanoma). Seven patients (24%) had stable disease. | Vonderheide et al., 2007 | DCR was not a formally reported endpoint and was not calculated for this table. |
| **Rüter et al., 2010** | CP-870,893 / selicrelumab | Conventional mAb; Systemic | advanced solid tumors (13 histologies; 41% melanoma) | Monotherapy | Phase I | Best overall response by RECIST v1.0; time to progression | No complete or partial response was observed. Stable disease was the best response in 7/ 27 patients (26%), with a median time to progression of 18 weeks; 20 patients had progressive | Rüter et al., 2010 | Time to progression was reported; formal PFS and OS estimates were not provided. |

| Study | Agent | Format and route | Tumor setting | Treatment context | Phase | Clinical activity endpoint(s) | Result as reported | Primary/ linked report(s) | Notes |
|---|---|---|---|---|---|---|---|---|---|
| | | | | | | | disease, with a median time to progression of 4.5 weeks. | | |
| **Beatty et al., 2013** | CP-870,893 / selicrelumab | Conventional mAb; Systemic | advanced pancreatic ductal adenocarcinoma (PDA), chemotherapy-naive; 90% metastatic | Chemotherapy combination (gemcitabine) | Phase I | ORR by RECIST v1.0; best overall response | ORR was 19%, comprising four partial responses. Eleven patients had stable disease. One patient who restarted gemcitabine alone after study withdrawal later had a partial response off study; that response was not included in the reported ORR. | Beatty et al., 2013; Wattenberg et al., 2021 | DCR was not identified as an author-defined endpoint and was not calculated. |
| **Vonderheide et al., 2013** | CP-870,893 / selicrelumab | Conventional mAb; Systemic | advanced solid tumors (mixed histologies; 78% metastatic melanoma) | Chemotherapy combination (carboplatin) | Phase I | ORR; best overall response by RECIST | Among 30 evaluable patients, six (20%) had partial responses, 12 (40%) had stable disease, and 12 (40%) had progressive disease; no complete response occurred. All partial responses occurred at 0.2 mg/ kg. Mean time from first partial response to progression was 75 days. | Vonderheide et al., 2013 | Schedule-specific results were not pooled into a new estimate. |
| **Nowak et al., 2015** | CP-870,893 / selicrelumab | Conventional mAb; Systemic | malignant pleural mesothelioma (MPM) | Chemotherapy combination (cisplatin + pemetrexed) | Phase Ib | Best radiological response by modified RECIST; PFS; OS | Six partial responses, eight cases of stable disease, and one case of progressive disease were reported among 15 treated patients. Median PFS was 6.3 months and median OS was 16.5 months. | Nowak et al., 2015; McDonnell et al., 2017 | The abstract reports nine cases of stable disease, whereas the Results section reports six partial responses, eight cases of stable disease, and one case of progressive disease among 15 patients; the internally consistent Results-section distribution is reported here. |
| **Bajor et al., 2018** | CP-870,893 / selicrelumab | Conventional mAb; Systemic | metastatic melanoma | ICI combination (tremelimumab) | Phase I | ORR; best overall response; OS | Among 22 evaluable patients, ORR was 27.3% (95% CI 8.7–45.9), comprising two complete responses | Bajor et al., 2018 | Clinical activity is presented descriptively as reported. |

| Study | Agent | Format and route | Tumor setting | Treatment context | Phase | Clinical activity endpoint(s) | Result as reported | Primary/ linked report(s) | Notes |
|---|---|---|---|---|---|---|---|---|---|
| | | | | | | | and four partial responses. Four of the six responses occurred at the MTD; nine patients survived for more than three years. | | |
| **Machiels et al., 2020** | CP-870,893 / selicrelumab | Conventional mAb; Systemic | advanced/ metastatic solid tumors (TNBC, ovarian, pancreatic, gastric, colorectal, melanoma, NSCLC, mesothelioma, other); most common: colorectal 29.7%, ovarian 18.9%, pancreatic 18.9% | Other combination (emactuzumab) | Phase Ib | Best overall confirmed response by RECIST v1.1 | No objective clinical response was observed. Stable disease was the best overall confirmed response in 15/ 37 patients (40.5%). Two patients had tumor-size decreases of 9% and 23%, respectively, but both subsequently progressed. | Machiels et al., 2020 | DCR was not recalculated from response components. |
| **Byrne et al., 2021** | CP-870,893 / selicrelumab | Conventional mAb; Systemic | Resectable pancreatic ductal adenocarcinoma (PDAC) | Chemotherapy combination (gemcitabine + nab-paclitaxel) | Phase I | ORR/ DCR: NA; DFS; OS | This was a resectable neoadjuvant pancreatic cancer study in which 15 patients underwent surgery. Clinical activity was reported through postoperative DFS and OS rather than RECIST response; no ORR or DCR was reported. | Byrne et al., 2021; Blise et al., 2024 | Neoadjuvant resectable setting; ORR and DCR are not applicable. |
| **Irenaeus et al., 2019** | mitazalimab / ADC-1013 | Conventional mAb; Intratumoral/ local | Advanced/ metastatic solid tumors (mixed: melanoma, renal cell, colorectal/ colon, rectal, ovarian, cholangiocarcinoma, lung, breast, peritoneal, esophageal SCC) | Monotherapy | Phase I | Best overall response by RECIST v1.1 and immune-related RECIST | No complete or partial response was reported. Stable disease was the best response in one patient by RECIST v1.1 and in two patients by immune-related RECIST. No formal ORR, DCR, PFS, or OS estimate was reported. | Irenaeus et al., 2019 | Intratumoral and intravenous administration cohorts were not combined into a new efficacy estimate. |
| **Moreno et al., 2023** | mitazalimab / ADC-1013 | Conventional mAb; Systemic | advanced/ refractory solid tumors (mixed; most frequent colorectal 13.7%, sarcoma 9.5%, melanoma 7.4%, pancreatic 7.4%, | Monotherapy | Phase I | Best overall response by RECIST v1.1 | One partial response was reported and lasted 5.6 months. Stable disease occurred in | Moreno et al., 2023 | Results are presented as reported; DCR was not recalculated. |

| Study | Agent | Format and route | Tumor setting | Treatment context | Phase | Clinical activity endpoint(s) | Result as reported | Primary/ linked report(s) | Notes |
|---|---|---|---|---|---|---|---|---|---|
| | | | thymoma 6.3%) | | | | 35/ 95 patients (36.8%), including nine with stable disease for at least six months; 43/ 95 (45.3%) had progressive disease and one patient (1.1%) was not evaluable. | | |
| **Van Laethem et al., 2024** | mitazalimab / ADC-1013 | Conventional mAb; Systemic | metastatic pancreatic ductal adenocarcinoma (mPDAC) | Chemotherapy combination (mFOLFIRINOX) | Phase I/ II | Confirmed ORR; best overall response; DOR | Confirmed ORR was 23/ 57 (40%), meeting the prespecified primary endpoint. Best overall response comprised one complete response (2%), 22 partial responses (39%), 22 cases of stable disease (39%), and 12 cases of progressive disease (21%). Median DOR was 12.5 months (95% CI 7.5–not estimable). | Van Laethem et al., 2024; Van Laethem et al., 2025 | The 2025 biomarker report is an updated analysis of the same OPTIMIZE-1 trial and does not represent additional patients. |
| **Kucukcelebi et al., 2025** | mitazalimab / ADC-1013 | Conventional mAb; Systemic | metastatic pancreatic ductal adenocarcinoma (PDAC) | Vaccine-based combination (MesoPher autologous dendritic cell vaccine) | Phase I | Best radiological response; PFS; OS | No objective radiological response was observed. At the first evaluation, eight of 16 patients (50%) had stable disease, six (38%) had progressive disease, and two (13%) could not be assessed. Median PFS and OS were reported separately for patients with and without baseline progressive disease. | Kucukcelebi et al., 2025 | DCR was not identified as an author-defined endpoint and was not calculated. |
| **O'Hara et al., 2021** | sotigalimab / APX005M | Conventional mAb; Systemic | metastatic pancreatic ductal adenocarcinoma | Chemotherapy with or without ICI combination (gemcitabine plus nab-paclitaxel, with or without nivolumab) | Phase Ib | ORR; DCR by cohort; PFS; OS | Investigator-assessed ORR by RECIST v1.1 was 14/ 24 (58%) in the dose-limiting-toxicity-evaluable population. DCR | O'Hara et al., 2021; Padrón et al., 2022 | Cohort-specific results are retained; linked PRINCE analyses were integrated within the same trial family. |

| Study | Agent | Format and route | Tumor setting | Treatment context | Phase | Clinical activity endpoint(s) | Result as reported | Primary/ linked report(s) | Notes |
|---|---|---|---|---|---|---|---|---|---|
| | | | | | | | was reported by cohort: 5/ 6 (83%) in B1, 5/ 6 (83%) in B2, 5/ 6 (83%) in C1, and 6/ 6 (100%) in C2. PFS, OS, and DCR were post hoc analyses. | | |
| **Weiss et al., 2021** | sotigalimab / APX005M | Conventional mAb; Systemic | advanced melanoma, NSCLC, or RCC resistant to anti-PD-1/ PD-L1 (12 melanoma, 1 NSCLC, 13 RCC) | Cabiralizumab-based combination, with or without nivolumab | Phase I | Best overall response by RECIST v1.1 | Among 26 patients, one had an unconfirmed partial response (4%), eight had stable disease (31%), 16 had progressive disease (62%), and one was unevaluable (4%). | Weiss et al., 2021 | The partial response was unconfirmed; it is not presented as a confirmed ORR. DCR was not author-defined and was not calculated. |
| **Weiss et al., 2024** | sotigalimab / APX005M | Conventional mAb; Systemic | Metastatic/ unresectable melanoma (cutaneous nonacral, acral lentiginous, mucosal, unknown subtype) with confirmed progression on anti-PD-1 | ICI combination (nivolumab) | Phase II | Confirmed ORR; best overall response; DOR | Confirmed ORR was 5/ 33 (15.2%; 90% CI 6.2–29.3), with five partial responses. Ten patients (30.3%) had stable disease and 18 (54.5%) had progressive disease. One additional radiologic partial response was unconfirmed; the confirmed responses had a reported DOR of at least 26 months. | Weiss et al., 2024 | The additional radiologic partial response was unconfirmed and is not included in confirmed ORR. |
| **Ko et al., 2025** | sotigalimab / APX005M | Conventional mAb; Systemic | Esophageal and gastroesophageal junction (GEJ) cancer (adenocarcinoma 76%, squamous cell carcinoma 24%); resectable T1-3 Nx, locally advanced | Neoadjuvant chemoradiotherapy combination | Phase II | ORR/ DCR: NA; pathologic complete response; major pathologic response; R0 resection | Pathologic complete response occurred in 11/ 29 efficacy-evaluable patients (37.9%); eight additional patients had a major pathologic response. R0 resection was achieved in 25/ 29 efficacy-evaluable patients undergoing surgery (86.2%). | Ko et al., 2025; Soto et al., 2024 | The abstract reports an R0 resection rate of 87.9%, whereas the explicit numerator and denominator in the Results and Table 4 are 25/ 29 (86.2%); the latter is reported here. |

| Study | Agent | Format and route | Tumor setting | Treatment context | Phase | Clinical activity endpoint(s) | Result as reported | Primary/ linked report(s) | Notes |
|---|---|---|---|---|---|---|---|---|---|
| **Bentebibel et al., 2026** | sotigalimab / APX005M | Conventional mAb; Intratumoral/ local | metastatic cutaneous or mucosal melanoma (ICB-naive, unresectable stage III-IV) | ICI combination (pembrolizumab) | Phase I/ II | ORR; DCR; best overall response by RECIST v1.1 | Among 32 treated patients, ORR was 15/ 32 (47%; 95% CI 30.9–63.6): five complete and ten partial responses. Fourteen patients had stable disease and three had progressive disease; DCR was 29/ 32 (91%). At the 10-mg RP2D, ORR was 12/ 24 (50%) and DCR was 22/ 24 (92%). | Bentebibel et al., 2026 | Injected and non-injected lesion responses were additionally reported by the authors and were not pooled across lesion categories here. |
| **Osorio et al., 2025** | 2141-V11 | Fc-engineered/ Fc-optimized mAb; Intratumoral/ local | advanced/ metastatic solid tumors with cutaneous/ sc/ lymph node metastasis (breast cancer 7/ 12, melanoma 3/ 12, renal cell carcinoma 2/ 12) | Monotherapy | Phase I | Confirmed ORR; confirmed DCR; DOR | Confirmed ORR was 2/ 12 (16.6%; 95% CI 2.5–55.6), comprising two complete responses, and confirmed DCR was 4/ 12 (33.3%; 95% CI 12.3–73.8). Tumor burden decreased in six of 12 patients. Median DOR among the two complete responders was 12 months. | Osorio et al., 2025 | Source: the peer-reviewed Cancer Cell report by Osorio et al., 2025 (doi:10.1016/ j.ccell.2025.07.013). |
| **Coveler et al., 2023** | SEA-CD40 | Fc-engineered/ Fc-optimized mAb; Systemic | advanced solid tumors AND lymphomas (mixed; 56 solid tumors part A + 11 lymphoma part C). Lymphomas: DLBCL, classical Hodgkin lymphoma, indolent/ follicular lymphoma | Mixed monotherapy/ combination | Phase I | Best response reported separately for solid-tumor and lymphoma cohorts | Solid tumors (44 efficacy-evaluable patients): one partial response and five patients with prolonged stable disease of approximately six months or longer. Lymphomas (seven efficacy-evaluable patients): one durable complete response and three cases of stable disease, including one lasting more than six months; four patients with | Coveler et al., 2023 | Solid-tumor and lymphoma results are kept separate; no combined ORR/ DCR or mixed solid-tumor safety denominator was generated. |

| Study | Agent | Format and route | Tumor setting | Treatment context | Phase | Clinical activity endpoint(s) | Result as reported | Primary/ linked report(s) | Notes |
|---|---|---|---|---|---|---|---|---|---|
| | | | | | | | DLBCL were not efficacy-evaluable because of rapid progression before response assessment. | | |
| **Luke et al., 2021** | ABBV-428 | Tumor-targeted/ bispecific CD40 agonist; Systemic | advanced solid tumors (dose escalation: mixed - pancreatic, sarcoma, cholangiocarcinoma, NSCLC, cervical, colorectal, HNSCC, small intestine, peritoneal, mesothelioma, ovarian, unknown primary; expansion: platinum-resistant ovarian cancer and epithelioid/ biphasic mesothelioma) | Monotherapy | Phase I | Best overall response | No objective response was observed among 53 evaluable patients. Stable disease was the best response in 25/ 53 (47%) overall; three patients with mesothelioma had stable disease for more than six months. At the RP2D, nine of 25 patients (36%) had stable disease and 16 (64%) had progressive disease. | Luke et al., 2021 | DCR was not recalculated from response components. |
| **Melero et al., 2026** | RO7300490 | Tumor-targeted/ bispecific CD40 agonist; Systemic | advanced and/ or metastatic solid tumors (NSCLC 23.8%, mesothelioma 17.5%, cervical SCC, TNBC, HCC, H&N SCC, melanoma, urothelial, SCLC, esophageal SCC, other) | Monotherapy | Phase I | ORR; DCR; PFS | No objective response was observed by RECIST v1.1. DCR was 31/ 73 (42.5%; 95% CI 31.0–54.6), all stable disease; 15 of the 31 cases of stable disease lasted at least 12 weeks. Median PFS was 50 days. | Melero et al., 2026 | Results are presented for the enrolled dose cohorts as reported; no cross-study comparison was performed. |
| **Tran et al., 2024** | MEDI5083 | Trimerized CD40L–IgG4 Fc-fusion protein; Systemic | advanced solid tumors (bladder n=3, breast n=2, colon n=5, NSCLC n=5, renal n=3, pancreatic n=4, SCCHN n=3) | ICI combination (durvalumab) | Phase I | ORR; disease control; DOR | Among 36 response-evaluable patients, ORR was 2.8% (95% CI 0.1–14.5), comprising one confirmed partial response. Eleven patients (30.6%) had stable disease, including one unconfirmed partial response; disease control lasting at least 24 weeks occurred in 8/ 36 (22.2%; 95% CI 10.1– | Tran et al., 2024 | Sequential and concurrent treatment cohorts are retained as reported; no new pooled efficacy measure was calculated. |

| Study | Agent | Format and route | Tumor setting | Treatment context | Phase | Clinical activity endpoint(s) | Result as reported | Primary/ linked report(s) | Notes |
|---|---|---|---|---|---|---|---|---|---|
| | | | | | | | 39.2). DOR for the confirmed response was 7.3 months. | | |
| **Steeghs et al., 2026** | MP0317 | FAP×CD40 DARPin-based construct; Systemic | advanced solid tumors (colorectal n=12, pancreatic n=9, mesothelioma n=6, NSCLC n=4, breast n=3, endometrial n=3, GIST n=2, ovarian n=2, other n=5) | Monotherapy | Phase I | ORR; best overall response; DCR | One patient with gastrointestinal stromal tumor had an unconfirmed partial response; unconfirmed ORR was 2% (90% CI 0.1–11.5). Stable disease occurred in 14 patients (30%), and DCR at the first tumor assessment was 33%. All patients had progressed or discontinued by day 180. | Steeghs et al., 2026 | The partial response was unconfirmed and is not presented as a confirmed ORR. |
| **Lakhani et al., 2025** | SL-172154 | Bispecific CD47 inhibitor/ CD40 agonist Fc-fusion protein; Systemic | platinum-resistant ovarian (70.4%), fallopian tube (14.8%), or primary peritoneal (14.8%) cancer | Monotherapy | Phase I | Best overall response | No objective response was reported; stable disease occurred in 6/ 27 patients. Disease control rate was not explicitly defined by the authors. | Lakhani et al., 2025 | Results are presented as reported; no patient-level disease control rate was recalculated. |
| **Drew et al., 2026** | SL-172154 | Bispecific CD47 inhibitor/ CD40 agonist Fc-fusion protein; Systemic | Platinum-resistant ovarian, primary peritoneal, or fallopian tube cancer (high-grade epithelial ovarian cancer) | Other combination (Mirvetuximab soravtansine) | Phase Ib | ORR and clinical benefit rate by cohort/ subgroup; PFS; OS | MIRV cohort: in the FRα-high subgroup, ORR was 13/ 39 (33.3%; 95% CI 19.1–50.2) and clinical benefit rate was 27/ 39 (69.2%); in the FRα-medium/ low subgroup, ORR was 4/ 26 (15.4%; 95% CI 4.4–34.9) and clinical benefit rate was 9/ 26 (34.6%). PLD cohort: 21 patients were treated and 20 were response-evaluable; ORR was 4/ 20 (20.0%; 95% CI 5.7–43.7) and | Drew et al., 2026 | Cohorts and FRα subgroups are reported separately; no cross-cohort ORR or disease-control estimate was generated. |

| Study | Agent | Format and route | Tumor setting | Treatment context | Phase | Clinical activity endpoint(s) | Result as reported | Primary/ linked report(s) | Notes |
|---|---|---|---|---|---|---|---|---|---|
| | | | | | | | clinical benefit rate was 9/ 20 (45.0%). | | |

**Table notes**

1. Clinical activity findings are presented descriptively as reported.
2. No efficacy meta-analysis or formal cross-study comparison was performed.
3. Linked reports were integrated within the corresponding trial family and were not counted as additional independent studies.
4. Results were not combined across distinct cohorts, doses, tumor types, or treatment regimens.
5. NR, not reported.
6. NA, not applicable to the study setting or endpoint structure.

**Supplementary Table S7. Reported immunological and pharmacodynamic context of included CD40 agonist trials.**

| Study | Agent | Format and route | Tumor setting | Treatment context | Immunological or pharmacodynamic domain | Main finding as reported | Pretreatment immune-related measurements | Immune–safety relationship | Linked report(s) | Notes |
|---|---|---|---|---|---|---|---|---|---|---|
| **Ninmer et al., 2026** | CDX-1140 | Conventional mAb; Systemic (subcutaneous/ intradermal administration) | High-risk melanoma (cutaneous, mucosal, or ocular primary sites); 11/ 22 (50%) ocular melanoma | Vaccine-based combination (melanoma helper peptide vaccine) | Vaccine-specific T-cell, antibody, and tumor immune responses | Ex vivo vaccine-specific CD4 T-cell responses were detected in six of 22 participants, and one participant showed a type-1 helper T-cell response to mutant BRAF. Tumor samples showed increased markers of mature dendritic cells, antigen-presenting-cell activation, and proliferating T cells after treatment. Antibody responses to vaccine antigens were reported in five of 22 participants; these observations were presented descriptively for the treated trial population and were not used for cross-study inference. | NR | Not evaluated | None | — |
| **Johnson et al., 2015** | ChiLob 7/ 4 | Conventional mAb; Systemic | CD40-expressing advanced solid tumors (mesothelioma, melanoma, NSCLC, colorectal, cervix, laryngeal squamous, esophageal/ pancreatic adenocarcinoma, thymic carcinoma) and diffuse large B-cell lymphoma | Monotherapy | B-cell, natural-killer-cell, cytokine, and dendritic-cell pharmacodynamics | ChiLob7/ 4 produced a dose-dependent fall in circulating B cells with CD21 shedding, a transient reduction in natural killer cells with activation-marker changes, and increases in MIP-1β and interleukin-12 at | NR | Not evaluated | None | — |

| Study | Agent | Format and route | Tumor setting | Treatment context | Immunological or pharmacodynamic domain | Main finding as reported | Pretreatment immune-related measurements | Immune–safety relationship | Linked report(s) | Notes |
|---|---|---|---|---|---|---|---|---|---|---|
| | | | | | | higher doses. No consistent rise in interleukin-2, interferon-γ, tumor necrosis factor, or interleukin-6 was observed, and dendritic-cell phenotypic changes were not consistent; these observations were presented descriptively for the treated trial population and were not used for cross-study inference. | | | | |
| **Vonderheide et al., 2007** | CP-870,893 / selicrelumab | Conventional mAb; Systemic | advanced solid tumors (melanoma, NSCLC, sarcoma, cholangiocarcinoma, breast, thyroid, unknown primary, mesothelioma) | Monotherapy | B-cell pharmacodynamics and cytokine timing | CP-870,893 caused rapid depletion and activation of circulating B cells, including increased CD86 expression. Serum tumor necrosis factor and interleukin-6 rose after infusion and were temporally associated with grade 1–2 cytokine-release syndrome. This is reported only as a descriptive temporal association; no causal relationship between cytokine changes and cytokine-release syndrome is inferred; these observations were presented descriptively for | NR | Grade 1–2 cytokine-release syndrome was temporally associated with post-infusion serum TNF-α and IL-6 elevations; descriptive temporal association only. | None | The cytokine/ CRS relationship is descriptive and temporal only. |

| Study | Agent | Format and route | Tumor setting | Treatment context | Immunological or pharmacodynamic domain | Main finding as reported | Pretreatment immune-related measurements | Immune–safety relationship | Linked report(s) | Notes |
|---|---|---|---|---|---|---|---|---|---|---|
| | | | | | | the treated trial population and were not used for cross-study inference. | | | | |
| **Rüter et al., 2010** | CP-870,893 / selicrelumab | Conventional mAb; Systemic | advanced solid tumors (13 histologies; 41% melanoma) | Monotherapy | B-cell, dendritic-cell, and T-cell pharmacodynamics | Repeated CP-870,893 dosing produced B-cell depletion with persistent CD86 and CD54 activation-marker expression. Modest dendritic-cell activation or depletion and declines in circulating T cells were observed in some patients treated at the maximum tolerated dose, while interferon-γ and perforin findings were inconsistent. The report did not formally connect these immune changes with adverse events; these observations were presented descriptively for the treated trial population and were not used for cross-study inference. | NR | Not evaluated | None | — |
| **Beatty et al., 2013** | CP-870,893 / selicrelumab | Conventional mAb; Systemic | advanced pancreatic ductal adenocarcinoma (PDA), chemotherapy-naive; 90% metastatic | Chemotherapy combination (gemcitabine) | B-cell/ cytokine pharmacodynamics and pretreatment inflammatory context | CP-870,893 caused rapid B-cell activation and depletion and transient increases in interleukins 6, 8, and 10. Tumor samples were macrophage- | Pretreatment neutrophil-to-lymphocyte ratio, neutrophils, interleukin-6, interleukin-8, serum amyloid A, C-reactive protein, and circulating immune-cell phenotypes were | Not evaluated | Wattenberg et al., 2021 | Pretreatment inflammatory profiling from the linked report is separated from on-treatment changes. |

| Study | Agent | Format and route | Tumor setting | Treatment context | Immunological or pharmacodynamic domain | Main finding as reported | Pretreatment immune-related measurements | Immune–safety relationship | Linked report(s) | Notes |
|---|---|---|---|---|---|---|---|---|---|---|
| | | | | | | dominant and did not show clear T-cell infiltration after treatment. The linked report measured pretreatment inflammatory and immune-cell features and described on-treatment changes in B cells, monocytes, dendritic-like populations, and CD4 T-cell activation; these observations were presented descriptively for the treated trial population and were not used for cross-study inference. | measured in the linked report. | | | |
| **Vonderheide et al., 2013** | CP-870,893 / selicrelumab | Conventional mAb; Systemic | advanced solid tumors (mixed histologies; 78% metastatic melanoma) | Chemotherapy combination (carboplatin) | B-cell activation, antigen-specific responses, and cytokines | Treatment produced rapid depletion and activation of circulating B cells, including increased CD86 expression, without a major consistent change in peripheral T-cell subsets. Occasional melanoma-antigen-specific responses were observed, and interleukin-6 and tumor necrosis factor typically peaked approximately two hours after infusion. These effects were reported | NR | Not evaluated | None | — |

| Study | Agent | Format and route | Tumor setting | Treatment context | Immunological or pharmacodynamic domain | Main finding as reported | Pretreatment immune-related measurements | Immune–safety relationship | Linked report(s) | Notes |
|---|---|---|---|---|---|---|---|---|---|---|
| | | | | | | descriptively as systemic pharmacodynamic observations; these observations were presented descriptively for the treated trial population and were not used for cross-study inference. | | | | |
| **Nowak et al., 2015** | CP-870,893 / selicrelumab | Conventional mAb; Systemic | malignant pleural mesothelioma (MPM) | Chemotherapy combination (cisplatin + pemetrexed) | B-cell pharmacodynamics and serial immune monitoring | The primary report showed rapid depletion and activation of circulating B cells after CP-870,893. The linked immunomonitoring report described cyclical changes in dendritic-cell and T-cell subsets across serial samples, with some changes potentially influenced by treatment timing or premedication; B-cell measures remained the clearest pharmacodynamic signal. Pretreatment dendritic-cell subsets and ICOS-positive CD8 T-cell proportions were also measured; these observations were presented descriptively for the treated trial population and | Pretreatment peripheral-blood samples measured BDCA-2+ dendritic-cell subsets and ICOS+ CD8 T-cell proportions in the linked immunomonitoring report. | Not evaluated | McDonnell et al., 2017 | Pretreatment measurements from the linked report are separated from longitudinal on-treatment changes. |

| Study | Agent | Format and route | Tumor setting | Treatment context | Immunological or pharmacodynamic domain | Main finding as reported | Pretreatment immune-related measurements | Immune–safety relationship | Linked report(s) | Notes |
|---|---|---|---|---|---|---|---|---|---|---|
| | | | | | | were not used for cross-study inference. | | | | |
| **Bajor et al., 2018** | CP-870,893 / selicrelumab | Conventional mAb; Systemic | metastatic melanoma | ICI combination (tremelimumab) | T-cell activation, tumor infiltration, and pretreatment inflammatory markers | CP-870,893 was associated with proliferating or reinvigorated T-cell features, increased tumor CD8 T-cell infiltration and programmed death-ligand 1 expression, and expansion of T-cell receptor clonotypes. Pretreatment soluble CD25, C-reactive protein, and lactate dehydrogenase were measured as systemic immune or inflammatory markers and evaluated in relation to overall survival. These pretreatment measures are reported separately from on-treatment changes; these observations were presented descriptively for the treated trial population and were not used for cross-study inference. | Pretreatment soluble CD25, C-reactive protein, and lactate dehydrogenase were measured as systemic immune/ inflammatory markers. | Not evaluated | None | — |
| **Machiels et al., 2020** | CP-870,893 / selicrelumab | Conventional mAb; Systemic | advanced/ metastatic solid tumors (TNBC, ovarian, pancreatic, gastric, colorectal, melanoma, NSCLC, mesothelioma, other); most common: colorectal 29.7%, | Other combination (emactuzumab) | Circulating and tumor immune-cell pharmacodynamics | Selicrelumab was followed by a transient reduction in circulating B cells, increased activated CD8 T cells around day | NR | Not evaluated | None | — |

| Study | Agent | Format and route | Tumor setting | Treatment context | Immunological or pharmacodynamic domain | Main finding as reported | Pretreatment immune-related measurements | Immune–safety relationship | Linked report(s) | Notes |
|---|---|---|---|---|---|---|---|---|---|---|
| | | | ovarian 18.9%, pancreatic 18.9% | | | 8, and reduced circulating monocytes. Tumor samples showed reductions in macrophage measures and, at higher doses, diminished CD8 T-cell density. Planned cytokine assessment in tumor biopsies was technically infeasible, limiting interpretation of local pharmacodynamic effects; these observations were presented descriptively for the treated trial population and were not used for cross-study inference. | | | | |
| **Byrne et al., 2021** | CP-870,893 / selicrelumab | Conventional mAb; Systemic | Resectable pancreatic ductal adenocarcinoma (PDAC) | Chemotherapy combination (gemcitabine + nab-paclitaxel) | Tumor and systemic immune remodeling | Neoadjuvant selicrelumab was associated with T-cell enrichment and increased mature dendritic cells in treated tumors, alongside reduced M2-like macrophage and fibrosis features. Systemically, activated T-cell features, CXCL10 and CCL22 changes, and T-cell receptor clonal expansion were reported. The linked analysis further described less T-cell exhaustion and | NR | Not evaluated | Blise et al., 2024 | The linked report was integrated within the same trial family and not counted independently. |

| Study | Agent | Format and route | Tumor setting | Treatment context | Immunological or pharmacodynamic domain | Main finding as reported | Pretreatment immune-related measurements | Immune–safety relationship | Linked report(s) | Notes |
|---|---|---|---|---|---|---|---|---|---|---|
| | | | | | | more effector T-cell features in association with longer disease-free survival; these observations were presented descriptively for the treated trial population and were not used for cross-study inference. | | | | |
| **Irenaeus et al., 2019** | mitazalimab / ADC-1013 | Conventional mAb; Intratumoral/ local | Advanced/ metastatic solid tumors (mixed: melanoma, renal cell, colorectal/ colon, rectal, ovarian, cholangiocarcinoma, lung, breast, peritoneal, esophageal SCC) | Monotherapy | Circulating B cells and soluble mediators | In patients, mitazalimab caused a transient decrease and subsequent recovery of circulating B cells together with increased B-cell CD86 expression. MCP-1, interleukin-6, and tumor necrosis factor increased mainly after higher doses or deep intrahepatic intratumoral administration, whereas peripheral T-cell and tumor immune trends were not consistent; these observations were presented descriptively for the treated trial population and were not used for cross-study inference. | NR | Not evaluated | None | Patient-derived clinical findings only. |
| **Moreno et al., 2023** | mitazalimab / ADC-1013 | Conventional mAb; Systemic | advanced/ refractory solid tumors (mixed; most frequent colorectal 13.7%, | Monotherapy | Circulating immune cells, soluble mediators, and | Mitazalimab caused transient reductions in circulating B, T, | NR | Not evaluated | None | — |

| Study | Agent | Format and route | Tumor setting | Treatment context | Immunological or pharmacodynamic domain | Main finding as reported | Pretreatment immune-related measurements | Immune–safety relationship | Linked report(s) | Notes |
|---|---|---|---|---|---|---|---|---|---|---|
| | | | sarcoma 9.5%, melanoma 7.4%, pancreatic 7.4%, thymoma 6.3%) | | immunogenicity | and natural killer cells followed by recovery, together with early chemokine peaks one to four hours after infusion. Cytokine increases were generally modest, and antidrug antibodies were detected in 10.8% of participants. The findings were described as pharmacodynamic and immunogenicity observations without a formal immune–safety analysis; these observations were presented descriptively for the treated trial population and were not used for cross-study inference. | | | | |
| **Van Laethem et al., 2024** | mitazalimab / ADC-1013 | Conventional mAb; Systemic | metastatic pancreatic ductal adenocarcinoma (mPDAC) | Chemotherapy combination (mFOLFIRINOX) | Circulating immune activation and tumor pharmacodynamics | Mitazalimab produced an early transient reduction in circulating immune-cell counts, followed within 24 hours by activation of B cells, monocytes, and dendritic cells and later activation of T and natural killer cells. The linked OPTIMIZE-1 analysis associated proliferating | NR | Not evaluated | Van Laethem et al., 2025 | The linked 2025 report belongs to the same OPTIMIZE-1 trial family. |

| Study | Agent | Format and route | Tumor setting | Treatment context | Immunological or pharmacodynamic domain | Main finding as reported | Pretreatment immune-related measurements | Immune–safety relationship | Linked report(s) | Notes |
|---|---|---|---|---|---|---|---|---|---|---|
| | | | | | | immune-cell populations with clinical outcomes and described immune-related changes in three paired tumor biopsies; these observations were presented descriptively for the treated trial population and were not used for cross-study inference. | | | | |
| **Kucukcelebi et al., 2025** | mitazalimab / ADC-1013 | Conventional mAb; Systemic | metastatic pancreatic ductal adenocarcinoma (PDAC) | Vaccine-based combination (MesoPher autologous dendritic cell vaccine) | Vaccine-specific T cells and tumor microenvironment remodeling | Treatment was associated with activated and vaccine-specific CD4 T-cell responses, including interferon-γ and granzyme B production. Paired tumor biopsies showed increased CD3- and CD8-positive T-cell infiltration and reduced stromal collagen after treatment. The immune findings were presented as descriptive evidence of vaccine and tumor microenvironment effects rather than as comparative efficacy measures; these observations were presented descriptively for the treated trial population and were not used | NR | Not evaluated | None | — |

| Study | Agent | Format and route | Tumor setting | Treatment context | Immunological or pharmacodynamic domain | Main finding as reported | Pretreatment immune-related measurements | Immune–safety relationship | Linked report(s) | Notes |
|---|---|---|---|---|---|---|---|---|---|---|
| | | | | | | for cross-study inference. | | | | |
| **O'Hara et al., 2021** | sotigalimab / APX005M | Conventional mAb; Systemic | metastatic pancreatic ductal adenocarcinoma | Chemotherapy with or without ICI combination (gemcitabine plus nab-paclitaxel, with or without nivolumab) | Pretreatment immune correlates and on-treatment systemic pharmacodynamics | On treatment, circulating naïve B cells decreased while plasmablasts, dendritic-cell activation, and activated or proliferating T-cell features increased. The linked PRINCE report identified pretreatment dendritic-cell, B-cell, CD4 T-cell, and tumor immune features associated with survival and reported increases in interferon-γ, CXCL9, and CXCL10 after treatment. Pretreatment measurements are distinguished from on-treatment pharmacodynamic changes in the adjacent column; these observations were presented descriptively for the treated trial population and were not used for cross-study inference. | Pretreatment cross-presenting and conventional dendritic cells, HLA-DR+CCR7+ B cells, differentiated/ type-1 CD4 T cells, and tumor CD4 T-cell infiltration were measured in the linked PRINCE report. | Not evaluated | Padrón et al., 2022 | Pretreatment measurements were separated from on-treatment pharmacodynamic changes. |
| **Weiss et al., 2021** | sotigalimab / APX005M | Conventional mAb; Systemic | advanced melanoma, NSCLC, or RCC resistant to anti-PD-1/ PD-L1 (12 melanoma, 1 NSCLC, 13 RCC) | Cabiralizumab-based combination, with or without nivolumab | Circulating mediators and paired tumor immune-cell density | Circulating cytokines and chemokines increased four to 24 hours after sotigalimab and generally returned to baseline before | NR | Not evaluated | None | — |

| Study | Agent | Format and route | Tumor setting | Treatment context | Immunological or pharmacodynamic domain | Main finding as reported | Pretreatment immune-related measurements | Immune–safety relationship | Linked report(s) | Notes |
|---|---|---|---|---|---|---|---|---|---|---|
| | | | | | | the second cycle. In a small paired-biopsy subset, no statistically significant change in CD4 T-cell, CD8 T-cell, or macrophage density was demonstrated. A limited pretreatment correlate was reported but did not constitute systematic pretreatment immune profiling for this audit; these observations were presented descriptively for the treated trial population and were not used for cross-study inference. | | | | |
| **Weiss et al., 2024** | sotigalimab / APX005M | Conventional mAb; Systemic | Metastatic/ unresectable melanoma (cutaneous nonacral, acral lentiginous, mucosal, unknown subtype) with confirmed progression on anti-PD-1 | ICI combination (nivolumab) | Acute circulating cytokine and chemokine responses | Plasma collected before and four hours after sotigalimab showed increased mediators consistent with dendritic-cell, natural-killer-cell, and T-cell activation, including tumor necrosis factor, interleukin-12, and interferon-γ-related signals. These changes were reported as acute systemic pharmacodynamic effects after treatment. The study did not | NR | Not evaluated | None | — |

| Study | Agent | Format and route | Tumor setting | Treatment context | Immunological or pharmacodynamic domain | Main finding as reported | Pretreatment immune-related measurements | Immune–safety relationship | Linked report(s) | Notes |
|---|---|---|---|---|---|---|---|---|---|---|
| | | | | | | formally evaluate whether these immune changes explained adverse events; these observations were presented descriptively for the treated trial population and were not used for cross-study inference. | | | | |
| **Ko et al., 2025** | sotigalimab / APX005M | Conventional mAb; Systemic | Esophageal and gastroesophageal junction (GEJ) cancer (adenocarcinoma 76%, squamous cell carcinoma 24%); resectable T1-3 Nx, locally advanced | Neoadjuvant chemoradiotherapy combination | Preoperative tumor microenvironment remodeling | A single preoperative dose was associated with increased abundance and activation of dendritic cells, monocytes, and macrophages, enhanced antigen-presentation programs, and greater cytotoxic T-cell infiltration and clonotype expansion in the tumor microenvironment. Regulatory T-cell features decreased in the linked translational analysis. The linked report evaluated a six-patient subset and was integrated with, rather than counted separately from, the parent trial. | NR | Not evaluated | Soto et al., 2024 | The linked translational analysis was a six-patient subset and was not counted as an additional independent trial. |
| **Bentebibel et al., 2026** | sotigalimab / APX005M | Conventional mAb; Intratumoral/ local | metastatic cutaneous or mucosal melanoma (ICB-naive, unresectable stage III- | ICI combination (pembrolizumab) | Local and systemic antigen-presenting-cell and T-cell | Intratumoral sotigalimab was followed by rapid activation | NR | Not evaluated | None | — |

| Study | Agent | Format and route | Tumor setting | Treatment context | Immunological or pharmacodynamic domain | Main finding as reported | Pretreatment immune-related measurements | Immune–safety relationship | Linked report(s) | Notes |
|---|---|---|---|---|---|---|---|---|---|---|
| | | | IV) | | activation | of dendritic cells and macrophages in injected tumors and early activation of circulating antigen-presenting cells. Later assessments showed T-cell activation, increased CD8 T-cell infiltration, and expansion of T-cell clonotypes in injected and non-injected tumors. The report interpreted these changes as evidence of local and systemic immune activation after treatment; these observations were presented descriptively for the treated trial population and were not used for cross-study inference. | | | | |
| **Osorio et al., 2025** | 2141-V11 | Fc-engineered/ Fc-optimized mAb; Intratumoral/ local | advanced/ metastatic solid tumors with cutaneous/ sc/ lymph node metastasis (breast cancer 7/ 12, melanoma 3/ 12, renal cell carcinoma 2/ 12) | Monotherapy | Systemic T-cell activation and tumor tertiary lymphoid structures | Patients with complete responses showed systemic CD8 T-cell activation and clonal expansion together with expansion of T follicular helper cells. Mature tertiary lymphoid structures were identified in tumor biopsies from complete | NR | Not evaluated | None | The source used is the peer-reviewed Cancer Cell article by Osorio et al., 2025 (doi:10.1016/ j.ccell.2025.07.013). |

| Study | Agent | Format and route | Tumor setting | Treatment context | Immunological or pharmacodynamic domain | Main finding as reported | Pretreatment immune-related measurements | Immune–safety relationship | Linked report(s) | Notes |
|---|---|---|---|---|---|---|---|---|---|---|
| | | | | | | responders, and higher doses were associated with increased inflammatory and interferon-γ-related circulating mediators. The findings were reported in the peer-reviewed Cancer Cell article, not treated as a separate preprint record. | | | | |
| **Coveler et al., 2023** | SEA-CD40 | Fc-engineered/ Fc-optimized mAb; Systemic | advanced solid tumors AND lymphomas (mixed; 56 solid tumors part A + 11 lymphoma part C). Lymphomas: DLBCL, classical Hodgkin lymphoma, indolent/ follicular lymphoma | Mixed monotherapy/ combination | Cytokines, immune-cell margination, and B-cell depletion | SEA-CD40 produced dose-dependent cytokine and chemokine changes, transient margination and activation of circulating immune cells, and cumulative depletion of B cells with repeated dosing. Persistent reductions in immunoglobulin concentrations were not observed. Tumor CD40 expression was evaluated but was not associated with the magnitude of tumor-burden reduction across the reported solid-tumor and lymphoma samples; these observations were presented descriptively for the treated trial population and | NR | Not evaluated | None | — |

| Study | Agent | Format and route | Tumor setting | Treatment context | Immunological or pharmacodynamic domain | Main finding as reported | Pretreatment immune-related measurements | Immune–safety relationship | Linked report(s) | Notes |
|---|---|---|---|---|---|---|---|---|---|---|
| | | | | | | were not used for cross-study inference. | | | | |
| **Luke et al., 2021** | ABBV-428 | Tumor-targeted/ bispecific CD40 agonist; Systemic | advanced solid tumors (dose escalation: mixed - pancreatic, sarcoma, cholangiocarcinoma, NSCLC, cervical, colorectal, HNSCC, small intestine, peritoneal, mesothelioma, ovarian, unknown primary; expansion: platinum-resistant ovarian cancer and epithelioid/ biphasic mesothelioma) | Monotherapy | Target occupancy and tumor/ circulating pharmacodynamics | Target occupancy exceeded 90% at doses of 0.8 mg/ kg or higher. Across available tumor samples, no consistent increase in intratumoral CD8 T cells, programmed death-ligand 1 expression, or immune-related transcriptional measures was observed, and circulating cytokine changes were minimal. The report described limited pharmacodynamic evidence beyond receptor occupancy and did not establish an immune–safety relationship; these observations were presented descriptively for the treated trial population and were not used for cross-study inference. | NR | Not evaluated | None | — |
| **Melero et al., 2026** | RO7300490 | Tumor-targeted/ bispecific CD40 agonist; Systemic | advanced and/ or metastatic solid tumors (NSCLC 23.8%, mesothelioma 17.5%, cervical SCC, TNBC, HCC, H&N SCC, melanoma, urothelial, SCLC, esophageal SCC, other) | Monotherapy | Tumor dendritic cells, tertiary lymphoid organization, and circulating B cells | Paired tumor biopsies showed increased DC-LAMP-positive dendritic cells and B cells, with structures consistent with early tertiary lymphoid | NR | Not evaluated | None | — |

| Study | Agent | Format and route | Tumor setting | Treatment context | Immunological or pharmacodynamic domain | Main finding as reported | Pretreatment immune-related measurements | Immune–safety relationship | Linked report(s) | Notes |
|---|---|---|---|---|---|---|---|---|---|---|
| | | | | | | organization. Circulating B cells decreased by approximately 90%, whereas meaningful cytokine elevation and consistent changes in intratumoral CD8 T cells or regulatory T cells were not observed. These findings were reported as pharmacodynamic evidence of CD40 pathway engagement; these observations were presented descriptively for the treated trial population and were not used for cross-study inference. | | | | |
| **Tran et al., 2024** | MEDI5083 | Trimerized CD40L–IgG4 Fc-fusion protein; Systemic | advanced solid tumors (bladder n=3, breast n=2, colon n=5, NSCLC n=5, renal n=3, pancreatic n=4, SCCHN n=3) | ICI combination (durvalumab) | Circulating B-cell and CD8 T-cell pharmacodynamics | Circulating B-cell counts decreased transiently on days 2–3 and recovered thereafter, while proliferating CD8 T cells increased around day 8. These cellular changes were reported as systemic pharmacodynamic effects of MEDI5083. Direct cytokine measurements and a formal analysis linking pharmacodynamic findings with | NR | Not evaluated | None | — |

| Study | Agent | Format and route | Tumor setting | Treatment context | Immunological or pharmacodynamic domain | Main finding as reported | Pretreatment immune-related measurements | Immune–safety relationship | Linked report(s) | Notes |
|---|---|---|---|---|---|---|---|---|---|---|
| | | | | | | cytokine-release syndrome were not reported in the study; these observations were presented descriptively for the treated trial population and were not used for cross-study inference. | | | | |
| **Steeghs et al., 2026** | MP0317 | FAP×CD40 DARPin-based construct; Systemic | advanced solid tumors (colorectal n=12, pancreatic n=9, mesothelioma n=6, NSCLC n=4, breast n=3, endometrial n=3, GIST n=2, ovarian n=2, other n=5) | Monotherapy | Tumor dendritic-cell activation, chemokines, and circulating B cells | At higher dose levels, paired tumor samples showed increased dendritic-cell abundance and a mature dendritic-cell transcriptional signature, accompanied by increased CXCL10. Circulating B cells showed a transient reduction and activation phenotype after dosing, while no clear or consistent increase in cytotoxic T-cell measures was demonstrated. The findings supported intratumoral and systemic pharmacodynamic activity without establishing an immune–safety relationship; these observations were presented descriptively for the treated trial | NR | Not evaluated | None | — |

| Study | Agent | Format and route | Tumor setting | Treatment context | Immunological or pharmacodynamic domain | Main finding as reported | Pretreatment immune-related measurements | Immune–safety relationship | Linked report(s) | Notes |
|---|---|---|---|---|---|---|---|---|---|---|
| | | | | | | population and were not used for cross-study inference. | | | | |
| **Lakhani et al., 2025** | SL-172154 | Bispecific CD47 inhibitor/ CD40 agonist Fc-fusion protein; Systemic | platinum-resistant ovarian (70.4%), fallopian tube (14.8%), or primary peritoneal (14.8%) cancer | Monotherapy | Target engagement, circulating cells, cytokines, and tumor macrophage/ T-cell changes | Near-complete target engagement was reported at doses of 3 mg/ kg or higher, with transient activation and margination of circulating B cells and monocytes and cyclical cytokine changes after dosing. Paired tumor biopsies at higher dose levels suggested a shift toward inflammatory macrophage features and increased granzyme B-positive CD8 T cells. These findings constitute the detailed monotherapy pharmacodynamic characterization of SL-172154. | NR | Not evaluated | None | — |
| **Drew et al., 2026** | SL-172154 | Bispecific CD47 inhibitor/ CD40 agonist Fc-fusion protein; Systemic | Platinum-resistant ovarian, primary peritoneal, or fallopian tube cancer (high-grade epithelial ovarian cancer) | Other combination (Mirvetuximab soravtansine) | Trial-specific reporting | No trial-specific immunological or pharmacodynamic findings were reported for the combination cohorts. | NR | Not evaluated | None | Detailed pharmacodynamic findings from the separate SL-172154 monotherapy trial were not attributed to this combination trial. |

**Table notes**

1. Immunological and pharmacodynamic findings are presented descriptively as reported.
2. No immunological/ pharmacodynamic meta-analysis or formal cross-study comparison was performed.
3. Linked reports were integrated within the corresponding trial family and were not counted as additional independent studies.
4. Pretreatment immune-related measurements were distinguished from on-treatment pharmacodynamic changes.
5. No causal relationship between immunological changes and adverse events should be inferred.
6. NR, not reported or not systematically identifiable.

7. NA, not applicable.

## Supplementary Figure S1. Forest plots, prediction intervals, and sensitivity analyses for the four exploratory proportional-synthesis pools.

**Supplementary Figure S1. Forest plots, prediction intervals, and sensitivity analyses for the four exploratory proportional-synthesis pools.**

**S1A. Primary random-effects analyses (PLOGIT, REML, Hartung–Knapp)**

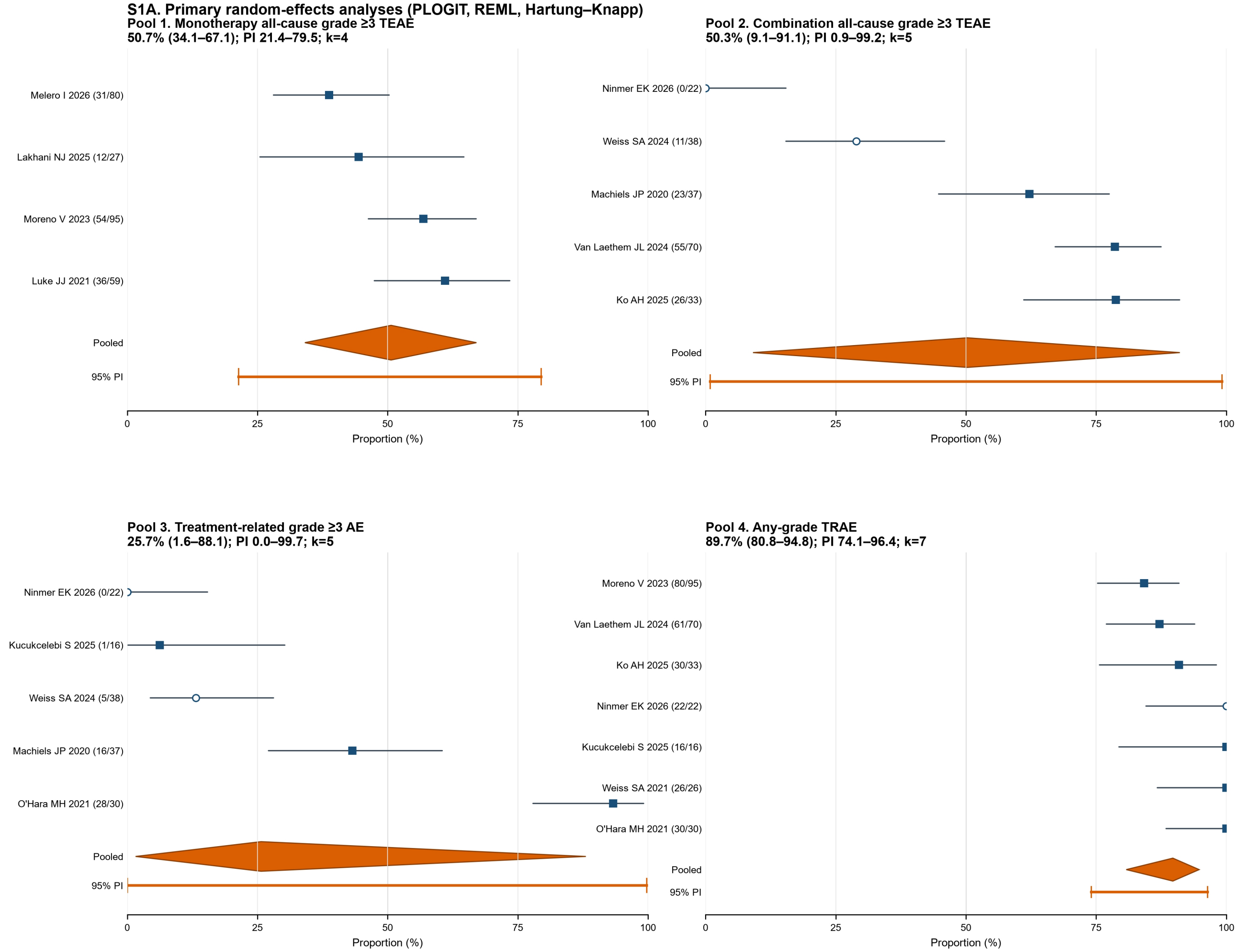


**S1B. Alternative transformation and fixed-effect analyses**

| Pool | Freeman–Tukey, % (95% CI) | Fixed effect, % (95% CI) |
|---|---|---|
| Pool 1 | 50.7 (33.9–67.4) | 51.0 (44.9–57.1) |
| Pool 2 | 46.8 (4.5–92.3) | 62.8 (54.7–70.2) |
| Pool 3 | 27.5 (0.0–83.5) | 35.3 (25.2–46.8) |
| Pool 4 | 95.9 (87.7–100.0) | 87.9 (83.1–91.5) |

Fixed-effect results are descriptive only and do not replace the primary random-effects analyses.

**S1C. GLMM boundary-study checks**

| Pool | k | GLMM estimate, % (95% CI) |
|---|---|---|
| Pool 1 | 4 | 50.7 (35.7–65.6) |
| Pool 2 | 5 | 43.0 (5.7–90.4) |
| Pool 3 | 5 | 19.2 (0.7–88.4) |
| Pool 4 | 7 | 96.7 (74.7–99.7) |

Binomial-normal GLMM estimates used no continuity correction.

**S1D. Derived-value exclusion analyses**

| Pool | Excluded input(s) | k | Estimate (95% CI) |
|---|---|---|---|
| Pool 1 | None | 4 | Not applicable |
| Pool 2 | U00013 and U00080 | 3 | Not available in locked sensitivity outputs |
| Pool 3 | U00013 and U00080 (all derived inputs) | 3 | Not available in locked sensitivity outputs |
| Pool 3* | U00080 (percentage-derived; locked analysis) | 4 | 29.4% (0.5–97.4) |
| Pool 4 | U00013 | 6 | Not available in locked sensitivity outputs |

Pool 3* is the available partial locked check (U00080 only). Requested all-derived Pool 2–4 estimates absent from locked outputs were not newly calculated.

Symbols: filled square, directly reported patient count; open circle, percentage- or narrative-derived count; orange diamond, pooled estimate; orange bar, 95% prediction interval. AE, adverse event; CI, confidence interval; GLMM, generalized linear mixed model; HK, Hartung–Knapp; PI, prediction interval; REML, restricted maximum likelihood; TEAE, treatment-emergent adverse event; TRAE, treatment-related adverse event.

S1A shows the locked primary random-effects proportional syntheses using logit-transformed proportions, restricted maximum-likelihood estimation, and Hartung–Knapp confidence intervals, with exact study-level 95% confidence intervals and 95% prediction intervals. Filled squares denote directly reported patient-level counts and open circles denote percentage- or narrative-derived counts; pooled estimates are shown as orange diamonds. S1B shows the locked Freeman–Tukey double-arcsine and descriptive fixed-effect sensitivity results. S1C shows the locked binomial-normal generalized linear mixed-model boundary-study checks without continuity correction. S1D summarizes derived-value exclusion analyses. A locked exclusion estimate was available only for the partial Pool 3 check after exclusion of U00080; requested all-derived exclusions for Pools 2–4 were not present in the locked outputs and were not newly calculated. No model, pool membership, event count, denominator, or locked estimate was changed.

Abbreviations: AE, adverse event; CI, confidence interval; GLMM, generalized linear mixed model; HK, Hartung–Knapp; PI, prediction interval; REML, restricted maximum likelihood; TEAE, treatment-emergent adverse event; TRAE, treatment-related adverse event.
