# Supplementary material for: Agonistic anti-CD40 antibodies in patients with solid tumors: a systematic review and evidence map of clinical safety, with exploratory proportional synthesis
Source: Front Immunol. 2026 Aug 27;17:1908586. doi: 10.3389/fimmu.2026.1908586 (PMC13558018; doi:10.3389/fimmu.2026.1908586)
Supplement: Supplementary file 2 [file Table1.docx]

**Table 1. Characteristics of the 27 independent primary safety reports included in the CD40 agonist safety evidence map.**

| **Study** | **Agent** | **Format** | **Route** | **Treatment context** | **Tumor type** | **Safety-evaluable N** | **Exploratory pool** |
| --- | --- | --- | --- | --- | --- | --- | --- |
| Ninmer et al., 2026 | CDX-1140 | Conventional mAb | Systemic (subcutaneous/intradermal administration) | Vaccine-based combination (melanoma helper peptide vaccine) | Melanoma | 22 | Yes (Pools 2/3/4) |
| Johnson et al., 2015 | ChiLob 7/4 | Conventional mAb | Systemic | Monotherapy | Basket/mixed solid tumors | 28 | No |
| Vonderheide et al., 2007 | CP-870,893 / selicrelumab | Conventional mAb | Systemic | Monotherapy | Basket/mixed solid tumors | 29 | No |
| Rüter et al., 2010 | CP-870,893 / selicrelumab | Conventional mAb | Systemic | Monotherapy | Basket/mixed solid tumors | 27 | No |
| Beatty et al., 2013 | CP-870,893 / selicrelumab | Conventional mAb | Systemic | Chemotherapy combination (gemcitabine) | PDAC | 21 | No |
| Vonderheide et al., 2013 | CP-870,893 / selicrelumab | Conventional mAb | Systemic | Chemotherapy combination (carboplatin) | Basket/mixed solid tumors | 32 | No |
| Nowak et al., 2015 | CP-870,893 / selicrelumab | Conventional mAb | Systemic | Chemotherapy combination (cisplatin + pemetrexed) | Mesothelioma | 15 | No |
| Bajor et al., 2018 | CP-870,893 / selicrelumab | Conventional mAb | Systemic | ICI combination (tremelimumab) | Melanoma | 24 | No |
| Machiels et al., 2020 | CP-870,893 / selicrelumab | Conventional mAb | Systemic | Other combination (emactuzumab) | Basket/mixed solid tumors | 37 | Yes (Pools 2/3) |
| Byrne et al., 2021 | CP-870,893 / selicrelumab | Conventional mAb | Systemic | Chemotherapy combination (gemcitabine + nab-paclitaxel) | PDAC | 16 | No |
| Irenaeus et al., 2019 | mitazalimab / ADC-1013 | Conventional mAb | Intratumoral/local | Monotherapy | Basket/mixed solid tumors | 23 | No |
| Moreno et al., 2023 | mitazalimab / ADC-1013 | Conventional mAb | Systemic | Monotherapy | Basket/mixed solid tumors | 95 | Yes (Pools 1/4) |
| Van Laethem et al., 2024 | mitazalimab / ADC-1013 | Conventional mAb | Systemic | Chemotherapy combination (mFOLFIRINOX) | PDAC | 70 | Yes (Pools 2/4) |
| Kucukcelebi et al., 2025 | mitazalimab / ADC-1013 | Conventional mAb | Systemic | Vaccine-based combination (MesoPher autologous dendritic cell vaccine) | PDAC | 16 | Yes (Pools 3/4) |
| O'Hara et al., 2021 | sotigalimab / APX005M | Conventional mAb | Systemic | Chemotherapy with or without ICI combination (gemcitabine plus nab-paclitaxel, with or without nivolumab) | PDAC | 30 | Yes (Pools 3/4) |
| Weiss et al., 2021 | sotigalimab / APX005M | Conventional mAb | Systemic | Cabiralizumab-based combination, with or without nivolumab | Basket/mixed solid tumors | 26 | Yes (Pool 4) |
| Weiss et al., 2024 | sotigalimab / APX005M | Conventional mAb | Systemic | ICI combination (nivolumab) | Melanoma | 38 | Yes (Pools 2/3) |
| Ko et al., 2025 | sotigalimab / APX005M | Conventional mAb | Systemic | Neoadjuvant chemoradiotherapy combination | Esophageal/GEJ cancer | 33 | Yes (Pools 2/4) |
| Bentebibel et al., 2026 | sotigalimab / APX005M | Conventional mAb | Intratumoral/local | ICI combination (pembrolizumab) | Melanoma | 32 | No |
| Osorio et al., 2025 | 2141-V11 | Fc-engineered/Fc-optimized mAb | Intratumoral/local | Monotherapy | Basket/mixed solid tumors | 12 | No |
| Coveler et al., 2023 | SEA-CD40 | Fc-engineered/Fc-optimized mAb | Systemic | Mixed monotherapy/combination | Mixed solid tumors and lymphomas | 67 | No |
| Luke et al., 2021 | ABBV-428 | Tumor-targeted/bispecific CD40 agonist | Systemic | Monotherapy | Basket/mixed solid tumors | 59 | Yes (Pool 1) |
| Melero et al., 2026 | RO7300490 | Tumor-targeted/bispecific CD40 agonist | Systemic | Monotherapy | Basket/mixed solid tumors | 80 | Yes (Pool 1) |
| Tran et al., 2024 | MEDI5083 | Trimerized CD40L–IgG4 Fc-fusion protein | Systemic | ICI combination (durvalumab) | Basket/mixed solid tumors | 38 | No |
| Steeghs et al., 2026 | MP0317 | FAP×CD40 DARPin-based construct | Systemic | Monotherapy | Basket/mixed solid tumors | 46 | No |
| Lakhani et al., 2025 | SL-172154 | Bispecific CD47 inhibitor/CD40 agonist Fc-fusion protein | Systemic | Monotherapy | Ovarian cancer | 27 | Yes (Pool 1) |
| Drew et al., 2026 | SL-172154 | Bispecific CD47 inhibitor/CD40 agonist Fc-fusion protein | Systemic | Other combination (Mirvetuximab soravtansine) | Ovarian cancer | 86 | No |

**Notes.**

Studies are grouped by biologic format and then ordered by agent, year, and first author. Reports in the non-monoclonal-antibody category are described using their specific construct formats.

Exploratory pool indicates that a report contributed an explicit denominator and a directly reported or reproducibly derived patient-level numerator to at least one prespecified exploratory proportional-synthesis pool: Pool 1, monotherapy all-cause grade ≥3 treatment-emergent adverse events; Pool 2, combination all-cause grade ≥3 treatment-emergent adverse events; Pool 3, treatment-related grade ≥3 adverse events; and Pool 4, any-grade treatment-related adverse events.

The SEA-CD40 report included patients with solid tumors and lymphomas; a solid-tumor-specific high-grade safety denominator could not be isolated.

Abbreviations: CD40L, CD40 ligand; DARPin, designed ankyrin repeat protein; Fc, fragment crystallizable; GEJ, gastroesophageal junction; ICI, immune checkpoint inhibitor; IgG4, immunoglobulin G4; mAb, monoclonal antibody; mFOLFIRINOX, modified folinic acid, fluorouracil, irinotecan, and oxaliplatin; PDAC, pancreatic ductal adenocarcinoma.
