# Supplementary material for: Agonistic anti-CD40 antibodies in patients with solid tumors: a systematic review and evidence map of clinical safety, with exploratory proportional synthesis
Source: Front Immunol. 2026 Aug 27;17:1908586. doi: 10.3389/fimmu.2026.1908586 (PMC13558018; doi:10.3389/fimmu.2026.1908586)
Supplement: Supplementary file 3 [file Table2.docx]

**Table 2. Safety-outcome reporting availability across the 27 independent primary safety reports.**

| **Safety outcome** | **Reports with explicit patient-level n/N** | **Limited aggregate or attribution-limited reporting** | **Per-event listing or unclear denominator** | **Not reported** | **Exploratory synthesis** |
| --- | --- | --- | --- | --- | --- |
| All-cause grade ≥3 TEAE | 10 | 0 | 17 (per-event only) | 0 | Pool 1 / Pool 2 |
| Treatment-related grade ≥3 AE | 12 | 15 (percentage only 2; all-cause only 8; treatment-related not grade-specific 5) | 0 | 0 | Pool 3 |
| Any-grade TRAE | 14 | 0 | 10 (per-event only) | 3 | Pool 4 |
| Cytokine-release reactions† | 16 | 0 | 2 (unclear denominator) | 9 | No |
| Infusion-related or hypersensitivity reaction | 15 | 0 | 2 (unclear denominator) | 10 | No |
| Hepatic toxicity | 16 | 0 | 8 (unclear denominator) | 3 | No |
| Serious adverse events | 12 | 0 | 2 (unclear denominator) | 13 | No |
| Treatment discontinuation due to toxicity | 16 | 0 | 1 (unclear denominator) | 10 | No |
| Treatment-related death | 19 | 0 | 0 | 8 | No |
| Dose-limiting toxicity | 22 | 0 | 2 (unclear denominator) | 3 | No |

**Notes.**

Values are numbers of reports; categories are mutually exclusive and sum to 27 for each outcome.

Reports with explicit patient-level n/N provided both a patient-level numerator and denominator. Limited aggregate or attribution-limited reporting comprised percentage-only reporting, all-cause reporting without treatment-related attribution, or treatment-related reporting without grade-specific patient-level data.

Per-event listings could not be summed to patient-level any-event counts because individual patients could experience multiple events. Patient-level cytokine-release-reaction counts were available in 16 reports but were not pooled because grading frameworks were heterogeneous or unspecified.

Dose-limiting toxicity was treated as a distinct dose-finding outcome and not as a substitute for treatment-related grade ≥3 adverse events. Pool definitions are provided in Table 3.

Abbreviations: AE, adverse event; DLT, dose-limiting toxicity; TEAE, treatment-emergent adverse event; TRAE, treatment-related adverse event.
