# Supplementary material for: Agonistic anti-CD40 antibodies in patients with solid tumors: a systematic review and evidence map of clinical safety, with exploratory proportional synthesis
Source: Front Immunol. 2026 Aug 27;17:1908586. doi: 10.3389/fimmu.2026.1908586 (PMC13558018; doi:10.3389/fimmu.2026.1908586)
Supplement: Supplementary file 4 [file Table3.docx]

**Table 3. Exploratory proportional synthesis of selected safety outcomes across CD40 agonist studies.**

| **Pool** | **Outcome** | **k** | **Events/N** | **Study-level range, %** | **Pooled proportion, % (95% CI)** | **I², %** | **Interpretive limitation** |
| --- | --- | --- | --- | --- | --- | --- | --- |
| Pool 1 | All-cause grade ≥3 TEAEs in monotherapy studies | 4 | 133/261 | 38.8–61.0 | 50.7 (34.1–67.1) | 66 | Monotherapy high-grade anchor; direct patient-level counts only; mixed biologic formats; limited by small k. |
| Pool 2 | All-cause grade ≥3 TEAEs in combination-therapy studies | 5 | 115/200 | 0–78.8 | 50.3 (9.1–91.1) | 89 | Very wide confidence interval; regimen-level estimate not attributable specifically to CD40 agonism. |
| Pool 3 | Investigator-reported treatment-related grade ≥3 AEs in the conventional/Fc-engineered systemic stratum | 5 | 50/143 | 0–93.3 | 25.7 (1.6–88.1) | 89 | Very wide confidence interval; investigator-attributed outcome with unresolved contribution of combination partners; all contributing reports evaluated conventional systemic antibodies. |
| Pool 4 | Any-grade treatment-related AEs | 7 | 265/292 | 84.2–100 | 89.7 (80.8–94.8) | 29 | Consistently high any-grade frequency with limited discrimination of high-grade toxicity. |

**Notes.**

Pooled proportions were estimated using inverse-variance random-effects models of logit-transformed proportions, with restricted maximum likelihood estimation and Hartung–Knapp adjustment; a continuity correction of 0.5 was applied where required. Estimates were exploratory and hypothesis-generating rather than comparative or confirmatory. Pools 2–4 included reproducibly derived inputs; sensitivity analyses and prediction intervals are provided in Supplementary Figure S1. k denotes the number of contributing reports.

Abbreviations: AE, adverse event; CI, confidence interval; I², percentage of total variability attributable to between-study heterogeneity; TEAE, treatment-emergent adverse event; TRAE, treatment-related adverse event.
